# Multispherical shapes of vesicles with intramembrane domains

**DOI:** 10.1140/epje/s10189-023-00399-z

**Published:** 2024-01-11

**Authors:** Reinhard Lipowsky

**Affiliations:** Max Planck Institute of Colloids and Interfaces, Science Park Golm, 14424 Potsdam, Germany

## Abstract

**Abstract:**

Phase separation of biomembranes into two fluid phases, *a* and *b*, leads to the formation of vesicles with intramembrane *a*- and *b*-domains. These vesicles can attain multispherical shapes consisting of several spheres connected by closed membrane necks. Here, we study the morphological complexity of these multispheres using the theory of curvature elasticity. Vesicles with two domains form two-sphere shapes, consisting of one *a*- and one *b*-sphere, connected by a closed *ab*-neck. The necks’ effective mean curvature is used to distinguish positive from negative necks. Two-sphere shapes of two-domain vesicles can attain four different morphologies that are governed by two different stability conditions. The closed *ab*-necks are compressed by constriction forces which induce neck fission and vesicle division for large line tensions and/or large spontaneous curvatures. Multispherical shapes with one *ab*-neck and additional *aa*- and *bb*-necks involve several stability conditions, which act to reduce the stability regimes of the multispheres. Furthermore, vesicles with more than two domains form multispheres with more than one *ab*-neck. The multispherical shapes described here represent generalized constant-mean-curvature surfaces with up to four constant mean curvatures. These shapes are accessible to experimental studies using available methods for giant vesicles prepared from ternary lipid mixtures.

**Graphic abstract:**

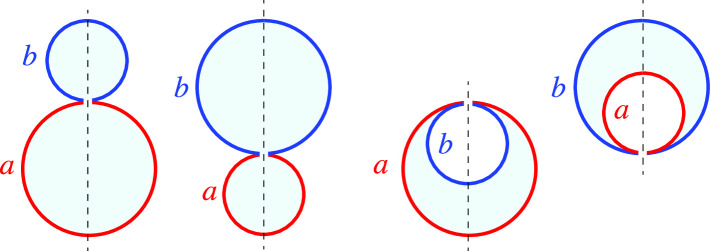

## Introduction

Biological and biomimetic membranes represent two-dimensional liquids. Biological membranes contain a large assortment of lipids and membrane proteins, whereas biomimetic membranes typically consist of a few lipid and protein components. These membranes should be able to undergo phase separation into two types of fluid domains, in close analogy to phase separation of liquid mixtures in three dimensions. This conclusion seems quite obvious from a theoretical point of view but, at the beginning of the 1990 s, it was rather difficult to find experimental evidence for it [1].

**Fig. 1:**
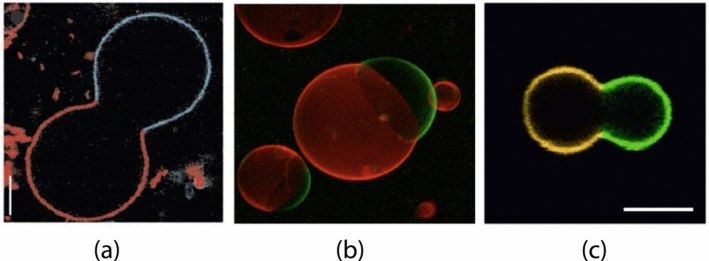
Domain-induced budding of giant unilamellar vesicles (GUVs) as predicted by theory [22, 23] and observed by fluorescence microscopy [4, 7, 10]. The two intramembrane domains consist of liquid-disordered (Ld) and liquid-ordered (Lo) lipid phases: **a** Cross-section through a vesicle that formed two lipid phase domains after a decrease in temperature. Reprinted with permission from Ref.  [4] (Copyright 2003, Springer-Nature); **b** Three-dimensional confocal scan of a two-domain vesicle that was formed by electrofusion. Reprinted with permission from Ref.  [7] (Copyright 2006, WSPC); and **c** Cross-section through a two-domain vesicle after osmotic deflation. Reprinted with permission from Ref.  [10] (Copyright 2021, Wiley) In each example, two different membrane dyes have been used to distinguish the Ld and Lo domains by fluorescence microscopy. The Ld phase is red in (**a**, **b**) and orange in (**c**), the Lo phase is blue in (**a**) and green in (**b**, **c**). Because the line tension of a domain boundary is positive, this boundary can reduce its line energy by constricting the vesicle *via* an open membrane neck. Scale bars: 5 $\mu$m in (**a**) and $10\,\mu$m in (**c**)

This situation has now changed completely because many ternary lipid mixtures have been identified which exhibit two coexisting fluid phases, see Fig. 1. Phase separation in ternary lipid mixtures has been observed for a variety of membrane systems including giant unilamellar vesicles (GUVs) [2–10], solid-supported membranes [11–13], hole-spanning (or black lipid) membranes [14], as well as pore-spanning membranes [15]. The phase diagrams of such three-component membranes have been determined using spectroscopic methods [16] as well as fluorescence microscopy of giant vesicles and X-ray diffraction of membrane stacks [9, 17–19]. Fluid–fluid coexistence has even been found in giant plasma membrane vesicles that contain a wide assortment of different lipids and proteins [20, 21].

Direct evidence for the formation of two types of fluid domains was provided by single particle tracking that showed that both phases exhibit relatively fast lateral diffusion [2]. In addition, using GUVs, several theoretical predictions [22–24] could be directly confirmed: the growth and coalescence of small domains into larger ones; domain-induced budding; and small shifts of the domain boundary away from the waist-line of the membrane neck.

**Fig. 2:**
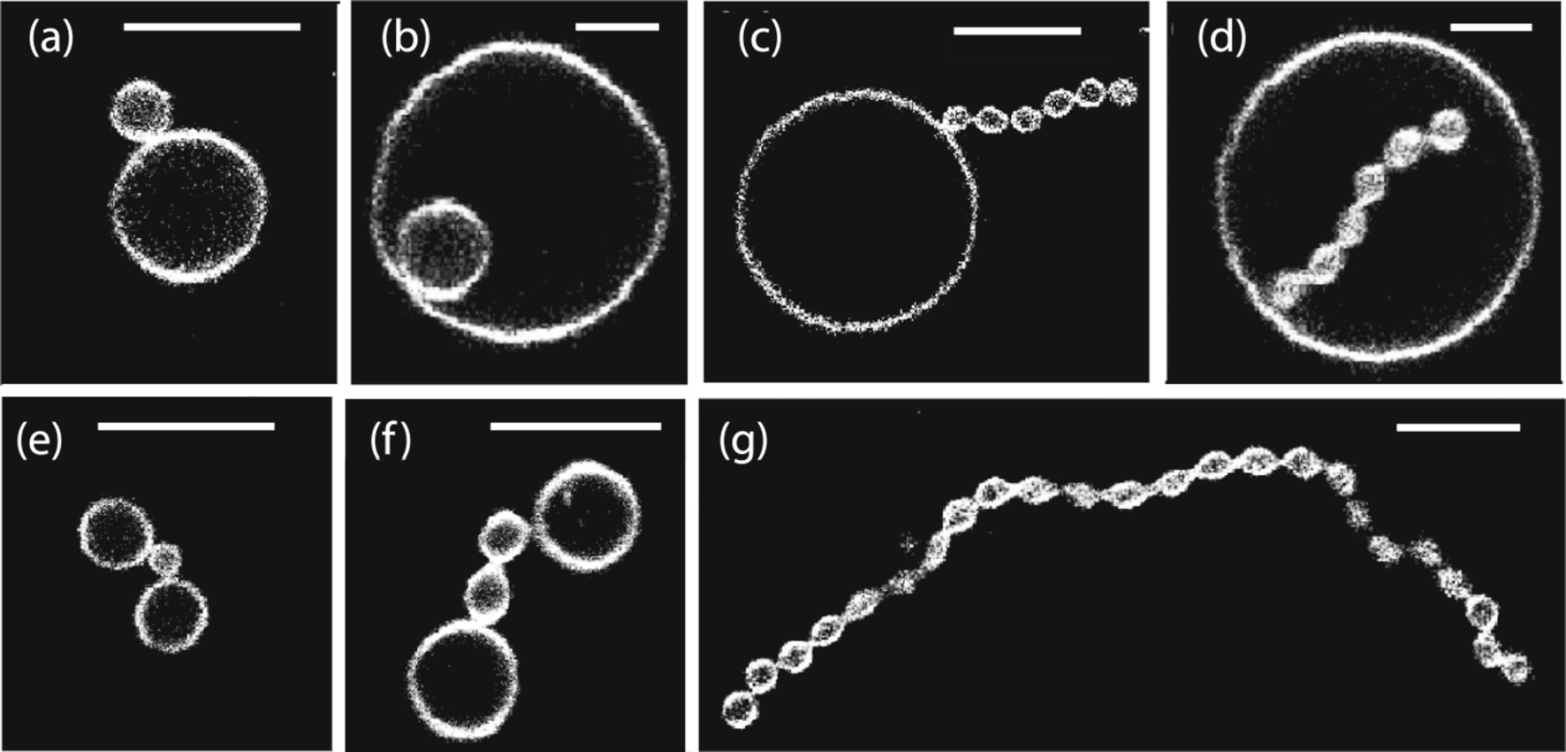
Multispherical shapes of GUVs with laterally uniform membranes: **a** Two-sphere shape consisting of one large and one small sphere, forming an out-bud; **b** Two-sphere shape with one large and one small sphere, forming an in-bud; **c** multisphere with one large sphere and a linear chain of six outward-pointing small spheres; **d** Multisphere with one large sphere and a linear chain of six inward-pointing small spheres; **e** Multisphere consisting of two large and one small spheres; **f** Multisphere with two large and two small spheres; and **g** Multisphere consisting of 24 equally sized spheres. In (**a**), (**c**), (**e**)–(**g**), all spheres have a positive mean curvature which implies that the membranes have a positive spontaneous curvature. In (**b**) and (**d**), the mean curvature of the small spheres is negative caused by a negative spontaneous curvature. All scale bars are 10 $\mu$m [25]

The three examples in Fig. 1 display vesicles with two intramembrane domains which are labeled by two different fluorophores. In all three examples, the boundary between the two domains forms an open membrane neck, which prefers to close when the volume of the vesicles is further reduced by osmotic deflation. Closed membrane necks can lead to a variety of multispherical shapes as observed for giant vesicles with laterally uniform membranes [25, 26]. In the latter case, the multispheres are built up from spheres with up to two different curvature radii, corresponding to large and small spheres, which are connected by closed membrane necks. Some examples for such multispherical shapes are displayed in Fig. 2. Each multisphere consists of large and small spheres but exhibits only two different radii, one for the large and one for the small spheres. More precisely, each large and small sphere is actually a punctured sphere that is connected to the punctures of neighboring spheres via closed membrane necks.

Here, the observed behavior of two-domain vesicles as illustrated in Fig. 1 and of multispherical vesicle shapes as found for uniform membranes, see Fig. 2, will be used to elucidate the morphological complexity of multispheres formed by vesicles with two or more intramembrane domains. The analysis is based on the theory of curvature elasticity. We consider different curvature-elastic properties of the two membrane domains apart from their Gaussian curvature moduli, which are taken to have identical values in the two domains. This simplifying assumption has several advantages. First, the domain boundaries between the intramembrane domains are located within the closed membrane necks [23]. Second, the multispherical shapes can be obtained by elementary calculus, without the need to use numerical methods for their computation. Third, identical Gaussian curvature moduli do not affect the vesicle shapes, which then depend on five membrane-elastic parameters as provided by two spontaneous curvatures and two bending rigidities as well as the line tension. Because the two bending rigidities are usually of the same order of magnitude, the morphological complexity of the multispheres depends primarily on the two spontaneous curvatures and on the line tension. At the end, we look at the changes arising from different Gaussian curvature moduli for the two domains. The most important change is related to the constriction forces at closed membrane necks as discussed in Sect. 10.2.

The paper is organized as follows: Section 2 provides a brief summary of multispherical shapes formed by uniform membranes and introduces the notion of positive and negative membrane necks. In Sect. 3, we will look at two-domain vesicles that form two-sphere shapes with a single *ab*-neck. Depending on the sign of the *ab*-neck and on the relative size of the *a*- and *b*-sphere, four two-sphere morphologies will be distinguished. The stability of these morphologies is governed by two stability relations, which apply to positive and negative *ab*-necks, respectively. Each closed *ab*-neck is subject to a constriction force that acts to compress the neck as described in Sect. 4. This constriction force depends primarily on the line tension of the domain boundary and on the spontaneous curvatures of the *a*- and *b*-domain. Large line tensions and/or large spontaneous curvatures generate constriction forces that drive the fission of the closed necks, whereas smaller line tensions and moderate spontaneous curvatures are unlikely to induce such a fission process.

The two-sphere shapes formed by two-domain vesicles are the simplest examples for multispheres of multi-domain vesicles. More complex morphologies are described in Sect. 5, corresponding to two-domain vesicles with multispheres formed by individual domains and to vesicles with more than two domains, which can transform into multispheres with more than one *ab*-neck. The stability regimes for multispheres with one *ab*-neck are determined in Sect. 6 and nested multispheres arising from nested domains are described in Sect. 7. The last two Sects. 8 and 9 interpret multispheres as generalized constant-mean-curvature surfaces and show how available methods for the experimental study of GUVs can be applied to multispheres with intramembrane domains. The changes arising from different Gaussian curvature moduli are described in Sect. 10.

## Multispherical shapes of uniform membranes

This section contains a brief review of the multispherical shapes as formed by uniform membranes, which are characterized by a uniform molecular composition and thus by uniform membrane-elastic parameters. A more detailed discussion of these shapes can be found in Ref. [26].

### Basic aspects of multispherical shapes

Each multisphere as displayed in Fig. 2 involves only a single fluid membrane, which encloses both the spherical compartments and the membrane necks connecting the spheres. Thus, each sphere is actually a punctured sphere, with its punctures being connected to the punctures of neighboring spheres via closed membrane necks. If we added a fluorescent probe to one spherical membrane segment, the probe would diffuse across the membrane necks and eventually spread over the whole multispherical,membrane. Likewise, the closed necks may undergo shape fluctuations, which lead to short-lived open necks, which transiently provide narrow water channels between the adjacent aqueous compartments.

Inspection of Fig. 2 reveals that each multisphere involves large and small spheres with only up to two different curvature radii, $R_l$ and $R_s$. These radii are intimately related to the mean curvatures $M_l$ and $M_s$ of the large and small spheres. As explained in the next subsection, the coexistence of two different sphere sizes on the same multispherical shape is a direct consequence of the shape equation for spherical membrane segments and implies that all spheres are subject to the same membrane tension and, thus, formed by a single membrane.

The multispherical shapes in Fig. 2 were experimentally observed to remain unchanged for many hours. This stability is primarily determined by the stability of the closed necks against neck opening. Stably closed necks require sufficiently large spontaneous curvatures of the vesicle membrane. As described in the next but one subsection, two stability conditions must be distinguished depending on the signs of the mean curvatures $M_l$ and $M_s$. The mean curvature $M_l$ of the large spheres is always positive, but the mean curvature $M_s$ of the small spheres can be positive or negative. Examples for small spheres with negative mean curvature, corresponding to inverted spheres, are provided by the small spheres in Fig. 2b, d.

### Local shape equation for uniform membranes

First, let us consider a membrane with uniform molecular composition that can be characterized by uniform spontaneous curvature *m* and uniform bending rigidity $\kappa$. When such a membrane forms a spherical segment, this segment attains a constant mean curvature *M* that satisfies the local shape (or Euler–Lagrange) equation [27]

1 $$\begin{aligned} {\Delta \!P}= 2\, \Sigma ^\textrm{tot}M - 4 \kappa m M^2 \,, \end{aligned}$$

in which the pressure difference

2 $$\begin{aligned} {\Delta \!P}\equiv P_\textrm{in}- P_\textrm{ex}\end{aligned}$$

between the interior and exterior aqueous solution is balanced by the linear term proportional to the total membrane tension

3 $$\begin{aligned} \Sigma ^\textrm{tot}\equiv \Sigma + 2 \kappa m^2 \, \end{aligned}$$

and by a second term, which is quadratic in the mean curvature *M*. Here, $\Sigma$ is the mechanical tension acting within the membrane and $2 \kappa m^2$ is the spontaneous tension [28] arising from the spontaneous curvature *m*.

Alternatively, the two parameters ${\Delta \!P}$ and $\Sigma$ can be viewed as two Lagrange multipliers used to minimize the bending energy for certain, prescribed values of the vesicle volume *V* and the membrane area *A*. For such a constrained minimization, ${\Delta \!P}$ and $\Sigma$ represent auxiliary variables that are conjugate to the geometric variables *V* and *A*. When we consider vesicles with a certain volume *V* and a certain membrane area *A*, the shape functional for these vesicles depends on the bending rigidity $\kappa$ and the spontaneous curvature *m* as well as on the two geometric parameters *V* and *A*. Using the bending rigidity as the basic energy scale and the vesicle size $R_\textrm{ve}= \sqrt{A/(4\pi )}$ as the basic length scale, the vesicle shapes are found to depend only on two dimensionless shape parameters, the volume-to-area ratio (or reduced volume) *v* which is proportional to $V/A^{3/2}$ and the rescaled spontaneous curvature ${\bar{m}}= m R_\textrm{ve}$ [29].

#### Case-by-case analysis of mean curvature

For zero spontaneous curvature, $m = 0$, the local shape equation in (1) reduces to ${\Delta \!P}= 2\, \Sigma M$, which has the same form as the classical Young–Laplace equation for liquid droplets. In this special case, the shape equation has the single solution or root

4 $$\begin{aligned} M = \frac{{\Delta \!P}}{2\, \Sigma } \quad (m = 0) \end{aligned}$$

for the mean curvature *M* of the spherical segment. For nonzero spontaneous curvature, $m \ne 0$, the local shape equation in (1) can be rewritten in the form

5 $$\begin{aligned} (M - \sigma )^2 + \delta - \sigma ^2 = 0 \end{aligned}$$

with the two parameter combinations

6 $$\begin{aligned} \sigma \equiv \frac{\Sigma ^\textrm{tot}}{4 \kappa m} \quad \textrm{and} \quad \delta \equiv \frac{\Delta P}{4 \kappa m} \,. \end{aligned}$$

Inspection of Eq. (5) directly shows that this equation has no (real-valued) solution or root for

7 $$\begin{aligned} \delta - \sigma ^2 > 0 \,; \end{aligned}$$

one degenerate (double) root as given by

8 $$\begin{aligned} M = \sigma \quad \textrm{for} \quad \delta - \sigma ^2 = 0 \,; \end{aligned}$$

and two different roots

9 $$\begin{aligned} M_{+} = \sigma + \left( \sigma ^2 - \delta \right) ^{1/2} \,. \end{aligned}$$

and

10 $$\begin{aligned} M_{-} = \sigma - \left( \sigma ^2 - \delta \right) ^{1/2} \,. \end{aligned}$$

for the parameter range

11 $$\begin{aligned} \delta - \sigma ^2 < 0 \,. \end{aligned}$$

#### Multispherical architectures for uniform membranes

In principle, the two parameter combinations $\sigma$ and $\delta$ as defined in Eq. (6) can be positive or negative, depending in particular on the sign of the spontaneous curvature *m*. A detailed analysis as described in Ref. [26] reveals, however, that physically meaningful solutions $M_+$ and $M_-$ are only obtained for two cases, I and II. Case I is characterized by

12 $$\begin{aligned} M_{+}> M_{-}> 0 \quad \text {for }\sigma> 0\text { and }\delta >0. \end{aligned}$$

In this case, the multispheres consist of large and small spheres, both of which have positive mean curvature. Furthermore, the two radii $R_{l}$ and $R_{s}$ of the large and small spheres are given by

13 $$\begin{aligned} R_{l} = \frac{1}{M_{-}} \quad \textrm{and} \quad R_{s} = \frac{1}{M_{+}} \,. \end{aligned}$$

Here and below, all radii are taken to be positive. Examples for Case I are provided by panels a, c, e, and f of Fig. 2. In the last panel g of this figure, we see an example for many equally sized spheres, corresponding to the doubly degenerate root in Eq. (8).

On the other hand, Case II is given by

14 $$\begin{aligned}{} & {} M_{+} > 0 \quad \textrm{and} \quad M_{-}< - M_{+}< 0 \quad \text {for }\nonumber \\{} & {} \sigma<0\text { and }\delta < 0, \end{aligned}$$

corresponding to one large sphere with positive mean curvature $M_+$ and inverted small spheres with negative mean curvature $M_-$. For case II, the curvature radii $R_l$ and $R_s$ of the large and small spheres have the form

15 $$\begin{aligned} R_{l} = \frac{1}{M_{+}} \quad \textrm{and} \quad R_{s} = - \frac{1}{M_{-}} \,. \end{aligned}$$

Examples for case II are shown in panels b and d of Fig. 2. For both cases I and II, the formation of a multispherical shape provides direct evidence that all spherical membrane segments experience the same mechanical tension $\Sigma$ and that the whole multisphere is formed by a single bilayer membrane.

### Closure of open membrane necks

The second ingredient from curvature elasticity that is necessary to understand multispherical shapes is the formation of closed membrane necks. Two-sphere shapes of uniform vesicle membranes were originally obtained as limit shapes of smoothly curved shapes with open necks [29, 30], using numerical methods applied to curvature models. These models describe the membranes as elastic surfaces, governed by certain curvature-elastic parameters. Uniform membranes as considered in the present section are characterized by curvature-elastic parameters, which are laterally uniform along the whole membrane, reflecting the uniform molecular composition of the membrane.

For axisymmetric shapes, the minimization of the shape functional leads to a set of ordinary differential equations [29]. The solutions of these equations form a discrete set of energy branches. Along each of these branches, the vesicle shape evolves smoothly as we vary one of the model parameters until we encounter a limit shape that can no longer be obtained by solving the differential equations. The two-sphere shapes considered here represent such limit shapes, which involve kinks of the membrane contours at the membrane necks and discontinuities of the mean curvature across this neck.

Furthermore, the geometry of a multispherical shape does *not* depend on the spontaneous curvature but only on the volume-to-area ratio *v* as well as on the number of large and small spheres [26]. As a consequence, the limit shapes continue to exist when the energy branches are further continued, keeping the multispherical geometry fixed but changing a single curvature-elastic parameter such as the spontaneous curvature. Even though the vesicle shape remains unchanged along this continuation, the bending energy of the vesicle changes because this energy depends on the curvature-elastic parameters.

When the vesicle forms an axisymmetric shape with an open neck, this neck has a finite radius $R_\textrm{ne}$, which represents the radius of the waist-line around the neck. When the neck closes, the radius $R_\textrm{ne}$ goes to zero which implies that the second principal curvature $C_{2,\textrm{wl}} = 1/R_\textrm{ne}$ parallel to the waist-line diverges. However, the mean curvature *M* remains finite on both sides of the neck. Therefore, the divergence of the second principal curvature must be canceled by another divergence arising from the first principal curvature $C_{1,\textrm{wl}}$, which is equal to the contour curvature. Furthermore, as the neck becomes closed, the mean curvature of the membrane attains two finite but different values on the two sides of the neck which implies that the mean curvature develops a discontinuity across the closed neck.

### Stability of closed membrane necks

Each closed neck provides a connection between two spherical membrane segments *i* and *j* with mean curvatures $M_i$ and $M_j$. The stability of such a closed neck is governed by a stability condition that involves the spontaneous curvature *m* of the adjacent membrane segments and the effective mean curvature of the closed neck as defined by [31]

16 $$\begin{aligned} M_{ij}^\textrm{eff}\equiv \frac{1}{2} \left( M_i + M_j \right) \,. \end{aligned}$$

Note that the neck curvature $M_{ij}^\textrm{eff}$ represents a purely geometric quantity. When the large and small spheres can be resolved by optical microscopy as in Fig. 2, the neck curvature $M_{ij}^\textrm{eff}$ can be directly deduced from the optical images. Therefore, this curvature represents an observable quantity.

The form of the stability condition depends on the sign of the effective neck curvature $M_{ij}^\textrm{eff}$. For *positive* neck curvature $M_{ij}^\textrm{eff}> 0$, the stability condition is given by:

17 $$\begin{aligned} m \ge M_{ij}^\textrm{eff}= \frac{1}{2} \left( M_i + M_j \right) > 0 \end{aligned}$$

which can only be fulfilled for a sufficiently large and *positive* spontaneous curvature *m*. For the multispheres displayed in panels a, c, e, and f of Fig. 2, all closed membrane necks have positive neck curvatures $M_{ij}^\textrm{eff}> 0$. Furthermore, if the multisphere consists of a chain of equally sized spheres as in panel g of Fig. 2, all membrane necks have the same neck curvature, which is positive as well.

For *negative* neck curvature $M_{ij}^\textrm{eff}< 0$, the stability condition has the form

18 $$\begin{aligned} m \le M_{ij}^\textrm{eff}= \frac{1}{2} \left( M_i + M_j \right) < 0 \end{aligned}$$

which requires a sufficiently large and *negative* spontaneous curvature *m*. For the multispheres displayed in panels b and d of Fig. 2, all closed membrane necks have negative neck curvatures $M_{ij}^\textrm{eff}< 0$.

The stability conditions for a closed membrane neck as given by Eqs. (17) and (18) are *local* in the sense that they depend only on the geometry and on the spontaneous curvature of the two membrane segments adjacent to the membrane neck. In particular, these stability conditions do *not* depend on the global morphology of the vesicle as characterized by its volume and surface area or by the number of large and small spheres formed by the vesicle [26].

### Positive and negative membrane necks

It will be convenient to characterize the membrane necks by the sign of their effective neck curvature and to distinguish positive from negative necks. By definition, a “positive neck” has a positive effective mean curvature $M_{ij}^\textrm{eff}> 0$, whereas a “negative neck” has a negative effective mean curvature $M_{ij}^\textrm{eff}< 0$. Thus, the multispheres shown in Fig. 2 involve only positive necks apart from those in panels b and d, which involve only negative necks. Using these definitions, we obtain an alternative characterization of the two cases I and II distinguished in Sect. . Indeed, multispheres belonging to case I have only positive membrane necks, whereas multispheres belonging to case II have only negative necks.

Inspection of the different examples in Fig. 2 shows that positive membrane necks connect two interior subcompartments, whereas negative membrane necks connect two exterior subcompartments. Therefore, positive necks can be regarded as interior necks and negative necks as exterior necks [26]. In the following, we will focus on the distinction between positive and negative necks and will only occasionally refer to the equivalent distinction between interior and exterior necks.

## Two-sphere shapes of two-domain vesicles

In this section, we go back to Fig. 1, which displays several examples of giant vesicles with two intramembrane domains, visualized by different fluorophores. The two domains are now distinguished by the domain labels *a* and *b*. In Fig. 1, the budding process is incomplete in the sense that each two-domain vesicle assumes a dumbbell shape with an open neck. Furthermore, in each example, the domain boundary between the *a* and *b* domains is located within this open neck. In order to close the neck, we now imagine to reduce the vesicle volume, which can be achieved experimentally by osmotic deflation. As a result of this deflation process, we obtain a two-sphere shape consisting of an *a*-sphere and a *b*-sphere, which are connected by a closed *ab*-neck.

In the following subsections, we will first demonstrate that the geometry of two-sphere shapes formed by two-domain vesicles is completely determined by the area fractions of the two domains. Second, we will examine the stability of the closed *ab*-neck and determine the stability and instability regimes. These regimes will be visualized by morphology diagrams, which are defined in terms of the spontaneous curvatures of the *a*- and *b*-domains.

### Basic geometry of two-domain vesicles

The geometry of a single vesicle with two domains is determined by the vesicle volume *V*, the surface area *A* of its membrane, and the area fractions of the two domains. The vesicle size $R_\textrm{ve}$ is defined in terms of the membrane area *A* and given by

19 $$\begin{aligned} R_\textrm{ve}\equiv \sqrt{A / (4 \pi )} \,, \end{aligned}$$

which represents the radius of a sphere with area *A* and is taken to provide the basic length scale of the vesicles. Likewise, the rescaled vesicle volume has the form

20 $$\begin{aligned} v \equiv \frac{V}{ \frac{4 \pi }{3} R_\textrm{ve}^3} = 6 \sqrt{\pi } \frac{V}{A^{3/2}} \end{aligned}$$

with $0 \le v \le 1$ where the limiting value $v = 1$ corresponds to a spherical shape of the vesicle.

Now, consider a vesicle as in Fig. 1 with one *a*-domain and one *b*-domain with surface areas $A_a$ and $A_b$. The total surface area *A* of the vesicle membrane is given by:

21 $$\begin{aligned} A = A_a + A_b \,, \end{aligned}$$

and the area fractions $\Phi _a$ and $\Phi _b$ of the two domains are defined by

22 $$\begin{aligned} \Phi _a\equiv \frac{A_a}{A_a+ A_b} \quad \textrm{and} \quad \Phi _b\equiv \frac{A_b}{A_a+ A_b} \end{aligned}$$

with $$\Phi _a + \Phi _b = 1$$.

### Geometry of two-sphere shapes with two domains

Two-sphere shapes consisting of one *a*-sphere and one *b*-sphere are the simplest multispherical shapes that can be formed by vesicles with two domains, with the domain boundary being located within the closed membrane neck between the two spheres. The geometry of such two-sphere shapes depends on the radius $R_{a}$ of the *a*-sphere and the radius $R_{b}$ of the *b* sphere. As before, all radii are taken to be positive.

**Fig. 3:**
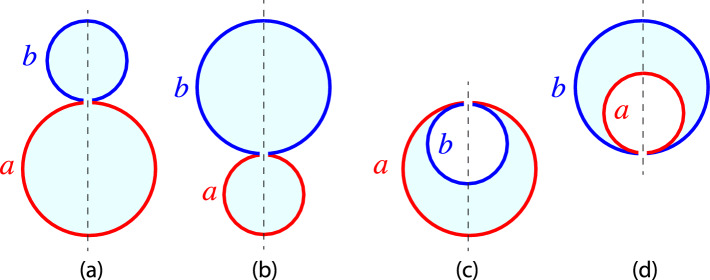
Different types of two-sphere shapes formed by vesicles with one *a*-domain (red) and one *b*-domain (blue). The radius of the *a*-sphere is denoted by $$R_{a}$$, the radius of the *b*-sphere by $$R_{b}$$: **a**, **b** Out-budded two-sphere shapes with $$R_{a} > R_{b} $$ in (**a**) and $$R_{b} > R_{a}$$ in (**b**); and (**c**, **d**) In-budded two-sphere shapes with $$R_{a} > R_{b} $$ in (**c**) and $$R_{b} > R_{a}$$ in (**d**) All four two-sphere vesicles have the same membrane area *A* but the vesicle volumes in (**a**, **b**) are larger than those in (**c**, **d**). The dashed vertical lines represent axes of rotational symmetry. The interior and exterior compartments are distinguished by cyan and white color, respectively

#### Radii and mean curvatures of two-sphere shapes

In general, the *a*-sphere may be larger than the *b*-sphere or vice versa as illustrated in Fig. 3. In addition, both spheres may have a positive mean curvature as in Fig. 3a, b or the smaller sphere may have a negative mean curvature as in Fig. 3c, d. Indeed, for the examples in Fig. 3a, b, the mean curvatures $M_{a}$ and $M_{b}$ of the *a*- and *b*-sphere are both positive and given by:

23 $$\begin{aligned} M_{a} = + \frac{1}{R_{a}}> 0 \quad \textrm{and} \quad M_{b} = + \frac{1}{R_{b}} > 0 \quad \text {(out)}\quad \end{aligned}$$

corresponding to *out-budded* two-sphere vesicles. On the other hand, for the example in Fig. 3c, which represents a two-sphere vesicle with an *in-bud* formed by the *b*-domain, these mean curvatures have the values

24 $$\begin{aligned} M_{a} = + \frac{1}{R_{a}} > 0 \quad \textrm{and} \quad M_{b}= - \frac{1}{R_{b}} <0 \quad \text {(in-}b\text {)}\quad \end{aligned}$$

whereas they are equal to

25 $$\begin{aligned} M_{a} = - \frac{1}{R_{a}} < 0 \quad \textrm{and} \quad M_{b} = + \frac{1}{R_{b}} > 0 \quad \text {(in-}a\text {)}\quad \end{aligned}$$

for the example in Fig. 3d, which displays a two-sphere vesicle with an *in-bud* formed by the *a*-domain.

#### Positive and negative *ab*-necks

Generalizing the definition for uniform membranes as given by Eq. (16), the effective mean curvature of the *ab*-necks is taken to be

26 $$\begin{aligned} M_{ab}^\textrm{eff}\equiv \frac{1}{2} \left( M_{a} + M_{b} \right) . \end{aligned}$$

where $M_a$ and $M_b$ are the mean curvature of the *a*- and *b*-sphere adjacent to the neck. Using this definition, the out-budded two-sphere shapes in Fig. 3a, b have a positive *ab*-neck with neck curvature

27 $$\begin{aligned} M_{ab}^\textrm{eff}\equiv \frac{1}{2} \left( \frac{1}{R_{a}} + \frac{1}{R_{b}} \right) > 0 \quad \text {(out)} \end{aligned}$$

as follows from Eq. (23). In contrast, the in-budded two-sphere shape in Fig. 3c with $R_{a} > R_{b}$ has a negative *ab*-neck with

28 $$\begin{aligned} M_{ab}^\textrm{eff}\equiv \frac{1}{2} \left( \frac{1}{R_{a}} - \frac{1}{R_{b}} \right) < 0 \quad \text {(in-}b\text {)} \,. \end{aligned}$$

Likewise, the shape in Fig. 3d with $R_{b} > R_{a}$ involves a negative *ab*-neck as well with effective neck curvature

29 $$\begin{aligned} M_{ab}^\textrm{eff}\equiv \frac{1}{2} \left( - \frac{1}{R_{a}} + \frac{1}{R_{b}} \right) < 0 \quad \text {(in-}a\text {)} \,. \end{aligned}$$

These effective neck curvatures will be useful to classify the different patterns of multispherical shapes as discussed further below.

For the out-budded two-spheres, the positive *ab*-neck provides a closed channel between two interior subcompartments. For the in-budded two-spheres, the negative *ab*-neck represents a closed channel between two exterior subcompartments. Thus, positive and negative *ab*-necks can again be regarded as interior and exterior necks in the sense, that interior *ab*-necks provide a connection between two interior subcompartments whereas exterior *ab*-necks connect two exterior subcompartments.

### Two-sphere geometry determined by area fractions

In terms of the surface areas $A_a$ and $A_b$ of the two domains, the radii of the *a*- and *b*-sphere are given by:

30 $$\begin{aligned} R_{a} = \sqrt{A_a/(4 \pi )} \quad \textrm{and} \quad R_{b} = \sqrt{A_b/(4 \pi )} \,. \end{aligned}$$

To simplify the mathematical formula, it will be convenient to define the rescaled radii

31 $$\begin{aligned} r_{a} \equiv \frac{R_{a}}{R_\textrm{ve}} \quad \textrm{and} \quad r_{b} \equiv \frac{R_{b}}{R_\textrm{ve}} \end{aligned}$$

with the vesicle size $R_\textrm{ve}= \sqrt{A/(4\pi )}$. For the two-sphere shapes formed by a two-domain vesicle as considered here, the rescaled radii become

32 $$\begin{aligned} r_{a} = \frac{\sqrt{A_a/(4 \pi )}}{\sqrt{A/(4 \pi )}} = \sqrt{\Phi _a} \end{aligned}$$

and

33 $$\begin{aligned} r_{b} = \frac{\sqrt{A_b/(4 \pi )}}{\sqrt{A/(4 \pi )}} = \sqrt{\Phi _b} \,. \end{aligned}$$

Furthermore, the rescaled and dimensionless mean curvatures

34 $$\begin{aligned} {\bar{M}}_{a} \equiv M_{a} R_\textrm{ve}\quad \textrm{and} \quad {\bar{M}}_{b} \equiv M_{b} R_\textrm{ve}\end{aligned}$$

are now given by

35 $$\begin{aligned} {\bar{M}}_{a} = \pm \frac{1}{r_{a}} = \pm \frac{1}{\sqrt{\Phi _a}} \quad \textrm{and} \quad {\bar{M}}_{b} = \pm \frac{1}{r_{b}} = \pm \frac{1}{\sqrt{\Phi _b}}\nonumber \\ \end{aligned}$$

where the plus and minus signs are determined by Eqs. (23)–(25), corresponding to the different two-sphere morphologies in Fig. 3.

The area decomposition in Eq. (21) now attains the simple form

36 $$\begin{aligned} 1 = r_{a}^2 + r_{b}^2 = \Phi _a + \Phi _b \,, \end{aligned}$$

which applies to both out-budded and in-budded two-sphere shapes. As far as the rescaled volume *v* is concerned, we have to distinguish three cases. For out-budded two-sphere vesicles as in Fig. 3a, b, the rescaled volume is given by

37 $$\begin{aligned} v = \frac{R_{a}^3 + R_{b}^3}{R_\textrm{ve}^3} = r_{a}^3 + r_{b}^3 = \Phi _a^{3/2} + \Phi _b^{3/2} \end{aligned}$$

with $\Phi _a = 1 - \Phi _b$. For in-budded two-sphere vesicles with the in-bud formed by the *b*-domain (Fig. 3c), the rescaled volume is

38 $$\begin{aligned} v = \frac{R_a^3 - R_b^3}{R_\textrm{ve}^3} = r_{a}^3 - r_{b}^3 = \Phi _a^{3/2} - \Phi _b^{3/2} \end{aligned}$$

Finally, when the in-bud is formed by the *a*-domain (Fig. 3d), the two-sphere vesicle has the rescaled volume:

39 $$\begin{aligned} v = \frac{R_{b}^3 - R_{a}^3}{R_\textrm{ve}^3} = r_{b}^3 - r_{a}^3 = \Phi _b^{3/2} - \Phi _a^{3/2} \,. \end{aligned}$$

Thus, all geometric properties of the two-sphere vesicles with one *a*-sphere and one *b*-sphere can be expressed in terms of the area fractions $\Phi _b$ and $\Phi _a = 1 - \Phi _b$.

**Fig. 4:**
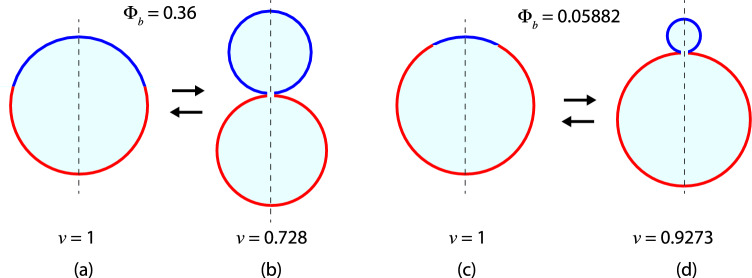
Formation of two-sphere shapes with positive *ab*-necks by osmotic deflation ($\rightarrow$), which reduces the vesicle volume whereas osmotic inflation ($\leftarrow$) increases this volume: **a**, **b** When deflated, a spherical two-domain vesicle with rescaled volume $v = 1$ and area fraction $\Phi _b = 9/25 = 0.36$ forms a two-sphere shape with one out-budded *a*-sphere of radius $r_a = 4/5$ and one out-budded *b*-sphere of radius $r_b = 3/5$, thereby reducing the rescaled volume from $v = 1$ to $v = 91/125 = 0.728$; and **c**, **d** Deflation of a spherical two-domain vesicle with rescaled volume $v = 1$ and area fraction $\Phi _b = 1/17 = 0.05882$ creates a two-sphere shape with one out-budded *a*-sphere of radius $r_a = 4/17^{1/2}$, one out-budded *b*-sphere of radius $r_b = 1/17^{1/2}$, and rescaled volume $v = 65/17^{3/2} = 0.9273$. Inflation of the two-sphere shapes in (**b**) and (**d**) leads back to the spherical two-domain vesicles in (**a**) and (**c**)

**Fig. 5:**
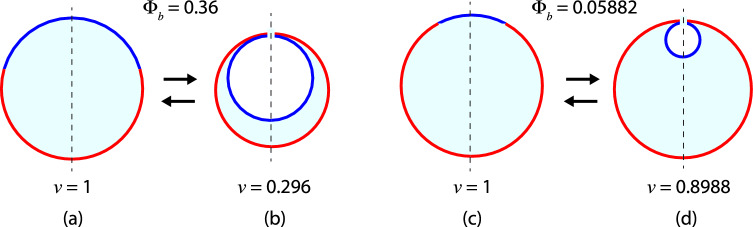
Formation of two-sphere shapes with an in-budded *b*-domain (blue) by osmotic deflation ($\rightarrow$), which reduces the vesicle volume whereas osmotic inflation ($\leftarrow$) increases this volume: **a**, **b** When deflated, a spherical two-domain vesicle with rescaled volume $v = 1$ and area fraction $\Phi _b = 9/25 = 0.36$ forms a two-sphere shape with one *a*-sphere of radius $r_a = 4/5$ and one in-budded *b*-sphere of radius $r_b = 3/5$, thereby reducing the rescaled volume to $v = 37/125 = 0.296$; and **c**, **d** Deflation of a spherical two-domain vesicle with rescaled volume $v = 1$ and area fraction $\Phi _b = 1/17 = 0.05882$ creates a two-sphere shape with one *a*-sphere of radius $r_a = 4/17^{1/2}$, one in-budded *b*-sphere of radius $r_b = 1/17^{1/2}$ and the rescaled volume $v = 63/17^{3/2} = 0.8988$. Inflation of the two-sphere shapes in (**b**) and (**d**) leads back to the single spheres in (**a**) and (**c**)

In order to illustrate the formation and characterization of two-sphere vesicles, we consider the examples in Figs. 4 and 5. We start from spherical vesicles with rescaled volume $v = 1$ and different area fractions $\Phi _b$. The vesicles are then exposed to an increased osmotic pressure in the exterior compartment, which acts to reduce the vesicle volume by osmotic deflation, a standard experimental procedure. Likewise, osmotic inflation can be applied to increase the vesicle volume. As a result of the deflation, the spherical vesicles may transform into out-budded two-sphere vesicles, for which both mean curvatures $M_{a}$ and $M_{b}$ are positive as in Fig. 4, or into two-sphere vesicles with an in-budded *b*-domain as in Fig. 5. The different cases of two-sphere vesicles with (i) $M_{a} > 0$ and $M_{b}>0$, (ii) $M_{a} > 0$ and $M_{b} < 0$, as well as (iii) $M_{a} < 0$ and $M_{b} > 0$, see Fig. 3, can be distinguished by different stability conditions for the closed *ab*-necks as described after the next subsection.

### Curvature discontinuities at domain boundary

The second ingredient from curvature elasticity that is necessary to understand the formation and stability of two-sphere vesicles is the stability of the closed *ab*-necks. The corresponding stability conditions for these necks are more involved than for uniform membranes. In fact, even for open necks, axisymmetric vesicles with two domains exhibit curvature discontinuities at the domain boundaries. These discontinuities can be computed explicitly for axisymmetric shapes parametrized by arc length *s*. The curvature discontinuities follow from the matching conditions for the mean curvatures $M_a(s_\textrm{db})$ and $M_b(s_\textrm{db})$ at the *a*- and *b*-sides of the domain boundary, which is located at arc length $s = s_\textrm{db}$.

These matching conditions are obtained from the first variation of the shape functional *F* as given by Eq. (A7) in Appendix A. The shape functional depends on the bending rigidity $\kappa _a$ and the spontaneous curvature $m_a$ of the *a*-domain as well as on the bending rigidity $\kappa _b$ and the spontaneous curvature $m_b$ of the *b*-domain. If we allowed the *a*- and *b*-domains to have different Gaussian curvature moduli $\kappa _{Ga}$ and $\kappa _{Gb}$, the first variation of the shape functional would lead to the matching condition [31]

40 $$\begin{aligned}{} & {} \kappa _a [ M_a(s_\textrm{db}) - m_a ] - \kappa _b [ M_b(s_\textrm{db}) - m_b ] \nonumber \\{} & {} \quad = \frac{1}{2} (\kappa _{Gb} - \kappa _{Ga}) \, C_2(s_\textrm{db}) \end{aligned}$$

where $C_2(s_\textrm{db})$ is the second principal curvature parallel to the domain boundary, which is continuous across this boundary.

For an axisymmetric dumbbell shape with an open neck, the second principal curvature $C_2(s_\textrm{db})$ is directly related to the neck radius $R_\textrm{ne}$ *via* $C_2(s_\textrm{db}) = 1/R_\textrm{ne}$. Therefore, this second principal curvature diverges if the neck radius vanishes. In order to avoid this divergence, the domain boundary moves away from the waist-line of the open neck during the neck closure process as shown by numerical calculations [23]. On the other hand, when the two Gaussian curvature moduli $\kappa _{Ga}$ and $\kappa _{Gb}$ have the same value, the matching condition in Eq. (40) simplifies and becomes

41 $$\begin{aligned} \kappa _a [ M_a(s_\textrm{db}) - m_a ] = \kappa _b [ M_b(s_\textrm{db}) - m_b ] \,, \end{aligned}$$

which is equivalent to the mean curvature discontinuity

42 $$\begin{aligned} \kappa _b M_b(s_\textrm{db}) - \kappa _a M_a(s_\textrm{db}) = \kappa _b m_b - \kappa _a m_a \end{aligned}$$

at the domain boundary. Thus, in contrast to the smoothly curved dumbbells formed by uniform membranes, the dumbbell shape of a two-domain vesicle exhibits a mean curvature discontinuity at the domain boundary as given by Eq. (42), even for $\kappa _{Ga} = \kappa _{Gb}$, that is, when both domains have the same Gaussian curvature modulus. Therefore, one should expect that the stability condition for a closed *ab*-neck is more complex than the corresponding condition for uniform membranes as shown in the next subsection.

### Stability of closed *ab* necks

The stability of closed *ab*-necks with respect to neck opening depends on the curvature-elastic parameters of the two membrane domains as provided by the spontaneous curvatures $m_a$ and $m_b$ as well as the bending rigidities $\kappa _a$ and $\kappa _b$ of the two domains. In addition, the stability of a closed *ab*-neck also depends on the line tension $\lambda$ of the domain boundary between the *a*- and *b*-domain.

In this subsection, we describe the stability conditions for the *ab*-necks of the different types of two-sphere shapes displayed in Fig. 3. The form of these conditions is somewhat different for the out-budded two-spheres in Fig. 3a, b, for the in-budded *b*-domains in Fig. 3c, and for the in-budded *a*-domains in Fig. 3d. These conditions can be visualized in terms of morphology diagrams that depend on the spontaneous curvatures $m_a$ and $m_b$ of the two membrane domains.

#### Neck stability for out-budded domains

The stability conditions for closed *ab*-necks can be obtained by looking at dumbbell shapes with slightly open necks and parametrizing these shapes by piece-wise constant-mean-curvature surfaces. For out-budded two-sphere vesicles, such a parametrization was first considered in Ref. [23] generalizing an analogous parametrization for uniform membranes in Ref. [32]. In this parametrization, one considers two hemispheres connected by an intermediate unduloid segment with neck radius $R_\textrm{ne}$. In the limit of small neck radius, the bending energy of the out-budded dumbbell shape behaves as:

43 $$\begin{aligned} E_\textrm{be}(R_\textrm{be}){} & {} = E_\textrm{be}(0) + R_\textrm{ne}\big [ 2 \pi \lambda + 4 \pi \big ( \kappa _a \left[ m_a - M_{a} \right] \nonumber \\{} & {} \quad + \kappa _b \left[ m_b - M_{b} \right] \big ) \big ] \end{aligned}$$

up to first order in the neck radius $R_\textrm{ne}$, with the mean curvatures $M_{a}$ and $M_{b}$ of the *a*- and *b*-sphere.^1^ The closed neck with $R_\textrm{ne}= 0$ is stable if the bending energy $E_\textrm{be}(R_\textrm{be})$ increases with increasing $R_\textrm{ne}$, that is, if [23]

44 $$\begin{aligned} \lambda /2 + \kappa _a \left( m_a - M_{a} \right) + \kappa _b \left( m_b - M_{b} \right) \ge 0 \,. \end{aligned}$$

This closed neck condition applies to both Fig. 3a, b, that is, to both a larger *a*-sphere with $$R_{a} > R_{b}$$ and to a larger *b*-sphere with $$R_{b} > R_{a}$$. A simple cross-check of the closed neck condition in Eq. (44) is obtained when we look at the limiting case of two identical domains with $$\kappa _a = \kappa _b$$, $$m_a = m_b$$, and $$\lambda = 0$$. In this limit, Eq. (44) reduces to $$2 m \ge M_{a} + M_{b}$$, the correct closed neck condition for uniform membranes as in Eq. (17) with $$M_i = M_{a} $$ and $$M_j = M_{b} $$.

*Stability condition in terms of rescaled variables* The stability condition in Eq. (44) becomes more transparent when we use the rescaled and dimensionless mean curvatures ${\bar{M}}_{a} = M_{a} R_\textrm{ve}= 1/r_{a}$ and ${\bar{M}}_{b} = M_{b} R_\textrm{ve}= 1/r_{b}$ as well as the rescaled and dimensionless spontaneous curvatures defined by

45 $$\begin{aligned} {\bar{m}}_a \equiv R_\textrm{ve}m_a \quad \textrm{and} \quad {\bar{m}}_b \equiv R_\textrm{ve}m_b \,. \end{aligned}$$

In terms of these rescaled variables, the stability condition in Eq. (44) becomes

46 $$\begin{aligned} \kappa _a \left( {\bar{m}}_a - \frac{1}{r_{a}} \right) + \kappa _b \left( {\bar{m}}_b - \frac{1}{r_{b}} \right) + \frac{\lambda R_\textrm{ve}}{2} \ge 0 \end{aligned}$$

This closed neck condition applies to both panels a and b of Fig. 3, that is, to $0 < \Phi _b \le 1/2$ as in Fig. 3a and to $1/2 \le \Phi _b < 1$ as in Fig. 3b. The line of limit shapes $L_{ab}$ is now described by the equality

47 $$\begin{aligned} \kappa _a \left( {\bar{m}}_a - \frac{1}{r_{a}} \right) + \kappa _b \left( {\bar{m}}_b - \frac{1}{r_{b}} \right) + \frac{\lambda R_\textrm{ve}}{2} = 0 \,. \end{aligned}$$

For the two-sphere vesicles discussed in the present section, the rescaled radii $r_{a}$ and $r_{b}$ can be expressed in terms of the area fractions $\Phi _a$ and $\Phi _b$ which leads to $r_{a} = \sqrt{\Phi _a}$ and $r_{b} = \sqrt{\Phi _b}$, see Eqs. (32) and (33).

**Fig. 6:**
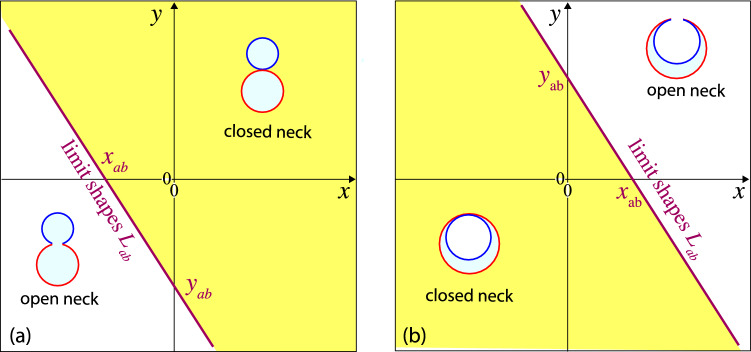
Stability regimes (yellow) for closed *ab*-necks of two-sphere vesicles within the morphology diagrams defined by the coordinates $x \equiv {\bar{m}}_a$ and $y \equiv {\bar{m}}_b$. All two-domain vesicles have the same area fraction $\Phi _b = 0.36$: **a** Stability regime for positive *ab*-necks of two-sphere vesicles with positive mean curvature $M_{a} >0$ of the *a*-sphere, positive mean curvature $M_{b}>0$ of the *b*-sphere, and positive effective mean curvature $M_{ab}^\textrm{eff}> 0$ of the *ab*-neck as in Eq. (27); and **b** Stability regime for negative *ab*-necks of two-sphere vesicles with in-budded *b*-domains (blue), corresponding to positive mean curvature $M_{a} >0$ of the *a*-sphere, negative mean curvature $M_{b}<0$ of the *b*-sphere, and negative effective mean curvature $M_{ab}^\textrm{eff}< 0$ of the *ab*-neck as in Eq. (28). In (**a**), all vesicles have constant volume $v = 0.728$ as in Fig. 4b. In (**b**), all vesicles have constant volume $v = 0.296$ as in Fig. 5b. In both panels, the line of limit shapes $L_{ab}$ (purple) separates vesicle shapes with stably closed necks from those with open necks. The intercepts of these $L_{ab}$-lines with the coordinate axes are denoted by $y_{ab}= {\bar{m}}_{b,ab}$ and $x_{ab} = {\bar{m}}_{a,ab}$. For out-budded shapes as in (**a**), these intercepts are given by Eqs. (50) and (51); for in-budded shapes as in (**b**), they are provided by Eqs. (59) and (60) further below

To visualize the stability regime for the closed *ab*-necks, it is convenient to rename the rescaled spontaneous curvatures and to define the coordinates

48 $$\begin{aligned} x \equiv {\bar{m}}_a \quad \textrm{and} \quad y \equiv {\bar{m}}_b \end{aligned}$$

for the two-dimensional morphology diagrams in Fig. 6. When Eq. (47) is solved for ${\bar{m}}_b = y$, the line of limit shapes $L_{ab}$ is described by the linear relation

49 $$\begin{aligned} y = h_\textrm{out}(x) \quad \textrm{with} \quad h_\textrm{out}(x) \equiv y_{ab} - \frac{\kappa _a}{\kappa _b} x \end{aligned}$$

and the intercept value

50 $$\begin{aligned} y_{ab} \equiv \frac{1}{r_{b}} + \frac{\kappa _a}{\kappa _b} \frac{1}{r_{a}} - \frac{\lambda R_\textrm{ve}}{2 \kappa _b} \approx - \frac{\lambda R_\textrm{ve}}{2 \kappa _b} \quad \text {(out).} \end{aligned}$$

The asymptotic equality ($\approx$) in Eq. (50) applies to giant vesicles with a large vesicle size $R_\textrm{ve}\gg \kappa _b/\lambda$.

In the (*x*, *y*)-plane, the line of limit shapes $L_{ab}$ as given by Eq. (49) is a straight line with negative slope $d y / dx = d {\bar{m}}_b /d {\bar{m}}_a = - \kappa _a/\kappa _b$, which intersects the *y*-axis at the intercept value $y_{ab}$, see Fig. 6a. Likewise, the $L_{ab}$-line intersects the *x*-axis at the intercept value

51 $$\begin{aligned} x_{ab} = \frac{\kappa _b}{\kappa _a} y_{ab} = \frac{\kappa _b}{\kappa _a} \frac{1}{r_{b}} + \frac{1}{r_{a}} - \frac{\lambda R_\textrm{ve}}{2 \kappa _a} \approx - \frac{\lambda R_\textrm{ve}}{2 \kappa _a} \quad \text {(out)}\nonumber \\ \end{aligned}$$

where the asymptotic equality again applies to giant vesicles with large size $R_\textrm{ve}$, which is implicitly assumed in Fig. 6a.

For a given value of the area fraction $\Phi _b = r_{b}^2$, the line of limit shapes $L_{ab}$ divides the (*x*, *y*)-plane into two parameter regimes corresponding to two-sphere vesicles with closed and with open *ab*-necks. As shown in Fig. 6a, the positive *ab*-neck is stably closed for

52 $$\begin{aligned} y \ge h_\textrm{out}(x) \quad \textrm{or} \quad {\bar{m}}_b \ge h_\textrm{out}({\bar{m}}_a) \,, \end{aligned}$$

but opens up for $y < h_\textrm{out}(x)$ or ${\bar{m}}_b < h_\textrm{out}({\bar{m}}_a)$, with the linear function $h_\textrm{out}(x)$ defined by Eqs. (49) and (50). The neck opens up in a continuous manner, that is, the neck radius $R_\textrm{ne}$ increases continuously from $R_\textrm{ne}= 0$ in the yellow stability regime above the $L_{ab}$-line in Fig. 6a to a nonzero value below this line.

#### Neck stability for in-budded domains

For two-sphere vesicles with in-budded *b*-domains as in Fig. 5b, the membrane shapes can again be parametrized by smoothly curved surface segments with piece-wise constant mean curvatures. In the limit of small neck radius $R_\textrm{ne}$, the bending energy of the in-budded shape then behaves as [31]

53 $$\begin{aligned} E_\textrm{be}(R_\textrm{be}){} & {} = E_\textrm{be}(0) + R_\textrm{ne}\big [ 2 \pi \lambda - 4 \pi \big ( \kappa _a \left[ m_a - M_{a} \right] \nonumber \\{} & {} \quad + \kappa _b \left[ m_b - M_{b} \right] \big ) \big ] \end{aligned}$$

up to first order in the neck radius $R_\textrm{ne}$. For two-sphere vesicles with an in-budded *b*-domain, the mean curvatures $M_{a}$ and $M_{b}$ of the *a*- and *b*-sphere are equal to $M_{a} = 1/R_{a}$ and $M_{b} = - 1/R_{b}$. Compared to the bending energy of the out-budded shape, see Eq. (43), the bending energy of the two-sphere vesicle with an in-budded *b*-domain as given by Eq. (53) involves two changes of sign. First, the mean curvature $M_{b} = - 1/R_{b}$ is now negative, whereas $M_{b}= + 1/R_{b}$ for out-budded shapes. In addition, the whole curvature-elastic term, which depends on the bending rigidities $\kappa _a$ and $\kappa _b$, is negative in Eq. (53), whereas it is positive in Eq. (43). The form of the closed neck condition in Eq. (53) does not change when we swap the domain labels *a* and *b* which implies that this closed neck condition also applies for in-budded *a*-domains.

The *ab*-neck of an in-budded *b*-domain is stably closed, if the bending energy $E_\textrm{be}(R_\textrm{ne})$ as given by Eq. (53) increases with increasing neck radius $R_\textrm{ne}$. Therefore, the closed *ab*-neck of an in-budded *b*-domain is stable if

54 $$\begin{aligned} \lambda / 2 - \big ( \kappa _a \left[ m_a - M_{a} \right] + \kappa _b \left[ m_b - M_{b} \right] \big ) \ge 0 \end{aligned}$$

which is equivalent to

55 $$\begin{aligned} \kappa _a \left[ m_a - M_{a} \right] + \kappa _b \left[ m_b - M_{b} \right] - \lambda / 2 \le 0 \,. \end{aligned}$$

A simple cross-check of this stability criterion is obtained for two identical domains with $\kappa _a = \kappa _b$, $m_a = m_b$, and $\lambda = 0$. In this case, Eq. (55) reduces to $2 m \le M_{a} + M_{b}$, the correct stability condition for uniform membranes as given by Eq. (18) with $M_i = M_{a}$ and $M_j = M_{b}$.

*Neck stability for in-budded* *b*-*domains* For an in-budded *b*-domain, the rescaled mean curvatures are given by ${\bar{M}}_{a} = M_{a} R_\textrm{ve}= + 1/r_{a}$ and ${\bar{M}}_{b} = M_{b} R_\textrm{ve}= - 1/r_{b}$. In terms of these rescaled curvatures, the stability condition in Eq. (55) becomes

56 $$\begin{aligned} \kappa _a \left( {\bar{m}}_a - \frac{1}{r_{a}} \right) + \kappa _b \left( {\bar{m}}_b + \frac{1}{r_{b}} \right) - \frac{\lambda R_\textrm{ve}}{2} \le 0 \,. \end{aligned}$$

In addition, an in-budded *b*-domain is only possible if the radius $r_{b}$ of the *b*-sphere does not exceed the radius $r_{a}$ of the *a*-sphere.

When the inequality in Eq. (56) becomes an equality, we obtain the line of limit shapes $L_{ab}$ for in-budded *b*-domains. Thus, for such *b*-domains, the line of limit shapes $L_{ab}$ is now given by

57 $$\begin{aligned} \kappa _a \left( {\bar{m}}_a - \frac{1}{r_{a}} \right) + \kappa _b \left( {\bar{m}}_b + \frac{1}{r_{b}} \right) - \frac{\lambda R_\textrm{ve}}{2} = 0 \,. \end{aligned}$$

For the two-sphere vesicles discussed in this section, the rescaled radii $r_{a}$ and $r_{b}$ are related to the area fractions $\Phi _a$ and $\Phi _b$ *via* $r_{a} = \sqrt{\Phi _a}$ and $r_{b} = \sqrt{\Phi _b}$. Using the previously introduced coordinates $x = {\bar{m}}_a$ and $y = {\bar{m}}_b$, the $L_{ab}$-line is described by

58 $$\begin{aligned} y = h_{b\text {-in}}(x) \quad \textrm{with} \quad h_{b\text {-in}}(x) \equiv y_{ab} - \frac{\kappa _a}{\kappa _b} x \end{aligned}$$

with the intercept value

59 $$\begin{aligned} y_{ab} \equiv -\frac{1}{r_{b}} + \frac{\kappa _a}{\kappa _b} \frac{1}{r_{a}} + \frac{\lambda R_\textrm{ve}}{2 \kappa _b} \approx \frac{\lambda R_\textrm{ve}}{2 \kappa _b} \quad \text {(}b\text {-in)} \end{aligned}$$

for the intersection of the $L_{ab}$-line with the *y*-axis, see the morphology diagram in Fig. 6b. Likewise, the $L_{ab}$-line intersects the *x*-axis at the intercept value

60 $$\begin{aligned} x_{ab} = \frac{\kappa _b}{\kappa _a} y_{ab} = - \frac{\kappa _b}{\kappa _a} \frac{1}{r_{b}} + \frac{1}{r_{a}} + \frac{\lambda R_\textrm{ve}}{2 \kappa _a} \approx \frac{\lambda R_\textrm{ve}}{2 \kappa _a} \,. \quad \text {(}b\text {-in)}\nonumber \\ \end{aligned}$$

For giant vesicles with a large value of $R_\textrm{ve}$, both intercepts $x_{ab}= {\bar{m}}_a^{ab}$ and $y_{ab} = {\bar{m}}_b^{ab}$ become large and positive as described by the asymptotic equalities in Eqs. (59) and (60), which is implicitly assumed in Fig. 6b. As shown in this figure, the *ab*-neck of an in-budded *b*-domain is stably closed for

61 $$\begin{aligned} y \le h_{b\text {-in}}(x) \quad \textrm{or} \quad {\bar{m}}_b \le h_{b\text {-in}}({\bar{m}}_a) \,, \end{aligned}$$

but opens up for $y > h_{b\text {-in}}(x)$ or ${\bar{m}}_b > h_{b\text {-in}}({\bar{m}}_a)$ with the linear function $h_{b\text {-in}}(x)$ defined in Eq. (58). The neck opens up in a continuous manner, that is, the neck radius $R_\textrm{ne}$ increases continuously from $R_\textrm{ne}= 0$ in the yellow stability regime below the $L_{ab}$-line in Fig. 6b to a nonzero value above this line.

For two-sphere vesicles with in-budded *a*-domains as in Fig. 3d, the rescaled mean curvatures are equal to ${\bar{M}}_{a} = - 1/r_{a}$ and ${\bar{M}}_{b} = + 1/r_{b}$. Therefore, the relationships for in-budded *a*-domains can be obtained from Eqs. (56), (57), (59), and (60), which have been derived for in-budded *b*-domains, by replacing $+1/r_{a}$ by $-1/r_{a}$ as well as $-1/r_{b}$ by $+1/r_{b}$ in all of these equations.

#### Out-budded versus in-budded domains

The line of limit shapes $L_{ab}$, which separates the stability regimes of the closed *ab*-necks from their instability regimes, corresponds to the purple lines in Fig. 6a, b. For two-sphere shapes with out-budded *b*-domains and positive *ab*-necks, the necks are stable above the purple $L_{ab}$-line in Fig. 6a. For in-budded *b*-domains and negative *ab*-necks, the necks are stable below the purple $L_{ab}$-line in Fig. 6b. For a fixed value of the area fraction $\Phi _b$, these two stability regimes exhibit a substantial overlap region, which is located between the purple line in Fig. 6a and the purple line in Fig. 6b, which are parallel to each other. Within this overlap region, the closed *ab*-necks are stable both for out-budded and for in-budded *b*-domains which implies the stability of both positive and negative *ab*-necks.

The two purple lines in Fig. 6a, b cross the *x*-axes at the two intercept values $x_{ab}$ as given by Eqs. (51) and (60). The difference between these two intercept values is:

62 $$\begin{aligned} x_{ab} \big |_ {b\text {-in}}- x_{ab} \big |_\textrm{out}= \frac{\lambda R_\textrm{ve}}{\kappa _a} - \frac{\kappa _b}{\kappa _a}\frac{2}{r_b} \approx \frac{\lambda R_\textrm{ve}}{\kappa _a} \,, \end{aligned}$$

where the asymptotic equality applies to giant vesicles with large $R_\textrm{ve}$-values. In such a situation, $\lambda R_\textrm{ve}/\kappa _a$ is of the order of $10^2$, which implies that the separation of the two lines of limit shapes $L_{ab}$ is quite large, leading to a broad overlap region.

The overlap region includes the parameter values close to the origin of the (*x*, *y*)-plane, corresponding to small spontaneous curvatures ${\bar{m}}_a$ and ${\bar{m}}_b$. Therefore, for small spontaneous curvatures, the *ab*-neck is stably closed both for out-budded and for in-budded *b*-domains. Consider, for instance, the two-domain vesicles in Figs. 4b and 5b, corresponding to area fraction $\Phi _b = 0.36$, which display an out-budded and in-budded *b*-domain, respectively. Thus, we predict that both two-sphere vesicles are stable for small spontaneous curvatures. Comparison of Figs. 4b with 5b also shows that these two-sphere vesicles have a rather different volume as given by $v = 0.728$ for the out-budded *b*-domain in Fig. 4b and by $v = 0.296$ for the in-budded *b*-domain in Fig. 5b. Therefore, reducing the volume of a spherical two-domain vesicle as in Fig. 4a will first lead to a two-sphere vesicle as in Fig. 4b with an out-budded *b*-domain. Further reduction of the volume may then transform the out-budded *b*-domain into an in-budded one as shown in Fig. 5b.

## Constriction forces and neck fission

The yellow stability regimes in Fig. 6 describe two-sphere shapes for a fixed value $\Phi _b = 0.36$ of the *b*-domain’s area fraction. As emphasized in Sect. 3.2, such a fixed value of the area fraction completely determines the geometry of the two-sphere vesicle, provided we distinguish out-budded from in-budded shapes. Thus, when we move across the yellow stability regimes in Fig. 6 by varying the spontaneous curvatures $x = {\bar{m}}_a$ and $y = {\bar{m}}_b$, we will always encounter the same two-sphere shape. However, such variations in the spontaneous curvatures have another important consequence: they change the constriction force acting against the closed neck. This constriction force is defined by

63 $$\begin{aligned} f \equiv \frac{\partial E_\textrm{be}}{\partial R_\textrm{ne}} \bigg |_{R_\textrm{ne}= 0} \ge 0 \end{aligned}$$

and represents the force acting against the closed neck.

### Constriction force for out-budded two-spheres

The yellow stability regime for out-budded two-sphere vesicles with fixed area fraction $\Phi _b = 0.36$ and rescaled volume $v = 0.728$ is displayed in Fig. 6a. Thus, when we move within this stability regime by changing the spontaneous curvatures ${\bar{m}}_a = x$ and ${\bar{m}}_b = y$ of the two membrane domains, the shape of the two-sphere vesicle remains unchanged. On the other hand, using the form of the bending energy $E_\textrm{be}$ as given by Eq. (43), the constriction force *f* as defined by Eq. (63) becomes

64 $$\begin{aligned} f = 2 \pi \lambda + 4 \pi \left[ \kappa _a \left( m_a - M_a \right) + \kappa _b \left( m_b - M_b \right) \right] \ge 0\nonumber \\ \end{aligned}$$

with $M_a = + 1/R_a$ and $M_b = +1/R_b$. This constriction force vanishes along the line of limit shapes $L_{ab}$, as described by Eq. (47) and illustrated in Fig. 6a. The force is positive within the yellow stability regime above the $L_{ab}$-line in Fig. 6a and increases with increasing line tension $\lambda$ as well as with increasing excess curvatures $m_a - M_a$ and $m_b - M_b$. The constriction force in Eq. (64) reduces to the particularly simple form

65 $$\begin{aligned} f = 2 \pi \lambda \end{aligned}$$

when the mean curvatures of the two spheres are equal to the spontaneous curvatures, that is, for

66 $$\begin{aligned} M_a = + \frac{1}{R_a} = m_a \quad \textrm{and} \quad M_b = + \frac{1}{R_b} = m_b \quad \text {(out)}\nonumber \\ \end{aligned}$$

corresponding to out-budded two-sphere vesicles with vanishing bending energy $E_\textrm{be}$ as follows from Eqs. (A2) and (A3).

### Constriction force for in-budded two-spheres

For in-budded *b*-domains, the limiting behavior of the bending energy $E_\textrm{be}$ for small neck radius $R_\textrm{ne}$ is given by Eq. (53). Using the definition ot the constriction force *f* in Equ (63), this force now becomes

67 $$\begin{aligned} f = 2 \pi \lambda + 4 \pi \big ( \kappa _a \left[ M_a - m_{a} \right] + \kappa _b \left[ M_b - m_{b} \right] \big ) \ge 0\nonumber \\ \end{aligned}$$

for closed necks of in-budded *b*-domains with $M_a = + 1/R_a$ and $M_b = -1/R_b$. The constriction force *f* in Eq. (67) vanishes along the line of limit shapes $L_{ab}$ as described by Eq. (57) and illustrated in Fig. 6b. The force is positive within the yellow stability regime below the $L_{ab}$-line in Fig. 6b and increases with increasing line tension $\lambda$ as well as with increasing excess curvatures $M_a - m_a$ and $M_b - m_b$.

For in-budded *a*-domains, the constriction force *f* has the same form as in Eq. (67) but with $M_a = - 1/R_a$ and $M_b = +1/R_b$. For both types of in-budded two-sphere shapes, the constriction force attains the simple form $f = 2 \pi \lambda$ when the mean curvatures are equal to the spontaneous curvatures, that is, for

68 $$\begin{aligned} M_a = + \frac{1}{R_a} = m_a \quad \textrm{and} \quad M_b = - \frac{1}{R_b} = m_b \quad ({b\text {-in}})\nonumber \\ \end{aligned}$$

or

69 $$\begin{aligned} M_a = - \frac{1}{R_a} = m_a \quad \textrm{and} \quad M_b = + \frac{1}{R_b} = m_b \quad ({a\text {-in}}) .\nonumber \\ \end{aligned}$$

In both cases, the in-budded two-sphere vesicles have vanishing bending energy.

### Fission of closed membrane necks

Sufficiently large constriction forces lead to the fission of closed membrane necks as observed experimentally for GUVs with uniform membranes [33]. More precisely, the closed necks of the GUVs were cleaved when the constriction forces exceeded about 20 pN. A similar threshold value for the constriction force is expected to apply to the two-domain vesicles considered here. Indeed, both for uniform and for two-domain membranes, the constriction force has to overcome an energy barrier provided by the formation of two ring-like bilayer edges across the closed membrane neck [27, 31].

For both out-budded and in-budded two-sphere vesicles, the constriction force includes the line tension term $2 \pi \lambda$, see Eqs. (64)–(67). The line tension $\lambda$ is equal to the excess free energy of the domain boundary per unit length. When the domain extends across both leaflets of the lipid bilayer, the domain boundary represents a cut through the whole bilayer. The cross-section of such a cut consists of three distinct regions: two hydrophilic headgroup regions with a combined thickness of about 1 nm and an intermediate hydrophobic tail region with a thickness of about 3 nm. For 3-dimensional fluid phases, a typical value for the interfacial free energy is of the order of 10 mN/m. If one assumes that this value is also applicable to the headgroup region of the lipid bilayer and that the latter region gives the main contribution to the line tension, one obtains the rough estimate $\lambda \simeq 10\,$pN. The latter value would lead to a contribution of about 63 pN to the constriction force *f*, which is equal to about three times the observed threshold value of 20 pN and, thus, sufficient to cleave the neck.

This simple estimate ignores the possible vicinity of a critical demixing point, at which the line tension must vanish. Therefore, close to such a critical point, the line tension can be reduced by orders of magnitude [22]. For the ternary mixture DOPC, sphingomyelin (SM), and cholesterol (CHOL), different compositions have been studied using giant vesicles. A detailed comparison of the experimentally observed two-domain shapes with the shapes computed in the framework of curvature elasticity [23] led to line tension values between 1 pN and 0.01 pN [4, 6, 8]. The line tension contribution $2 \pi \lambda$ to the constriction force then varies between 6.3 pN and 0.063 pN.

The other contributions to the constriction forces *f* in Eqs. (64) and (67) are proportional to the bending rigidities $\kappa _a$ and $\kappa _b$ as well as to the excess curvatures $\pm (M_a - m_a)$ and $\pm (M_b - m_b)$. The bending rigidities are of the order of $10^{-19}$ J. For giant vesicles, the excess curvatures are dominated by the spontaneous curvatures $m_a$ and $m_b$. Moderate spontaneous curvatures as generated by sugar asymmetries [25] are of the order of $1/(\mu$m). Larger spontaneous curvatures up to about $10/(\mu$m) can be obtained by the binding of His-tagged GFP to the outer membrane leaflet [33]. Therefore, the excess curvature terms contribute about 1 pN for $m_a \simeq m_b \simeq 1/(\mu$m) and about 10 pN for $m_a \simeq m_b \simeq 10/(\mu$m) to the constriction forces.

Combining the line tension contribution with the excess curvature contributions, we conclude that the constriction forces are sufficiently large to cleave the closed membrane neck when the line tension $\lambda \gtrsim$ 1 pN and the spontaneous curvatures are of the order of $10/(\mu$m). On the other hand, line tensions below 0.1 pN and moderate spontaneous curvatures of the order of $1/(\mu$m) are unlikely to induce neck fission.

## Morphological complexity of multispheres

The two-sphere shapes formed by two-domain vesicles as discussed in the previous section represent the simplest examples for multispheres that can be formed by vesicles with several membrane domains. In general, more complex shapes are also possible. First, each domain of a two-domain vesicle can form a multispherical shape itself. Second, vesicles with several *a*- and/or *b*-domains can attain multispheres with several *ab*-necks.

### Multispheres of two-domain vesicles

**Fig. 7:**
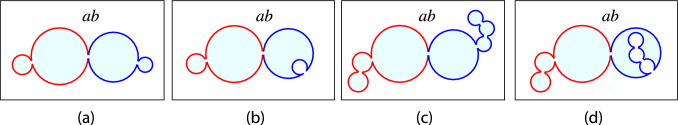
Some examples for multspherical shapes formed by two-domain vesicles. Each multisphere has a single *ab*-neck that contains the domain boundary between the *a*- and *b*-domain: **a**, **b** Four-sphere shapes with two *a*- and two *b*-spheres connected by one *aa*-neck and one *bb*-neck; and **c**, **d** Seven-sphere shapes with three *a*- and four *b*-spheres connected by two *aa*-necks and three *bb*-necks. In the examples displayed here, the *a*- and *b*-sphere connected by the *ab*-neck have positive mean curvatures which implies that each *ab*-neck is positive. Multispheres with negative *ab*-necks are discussed further below, see Sect. 6.2 and Fig. 11

For a two-domain vesicle, both the *a*- and the *b*-domains can attain a multispherical shape. When the *a*-domain transforms into a multispherical shape, this shape consists of two or more (punctured) *a*-spheres, which are connected by closed *aa*-necks. Likewise, when the *b*-domain forms a multispherical shape, this shape consists of two or more (punctured) *b*-spheres, which are connected by closed *bb*-necks. Thus, each multispherical shape formed by a two-domain vesicle involves both a single *ab*-neck as discussed in the previous Sect. 3 and additional membrane necks between two *a*-spheres or between two *b*-spheres, which are governed by the stability conditions for uniform membranes as described in Sect. 2.4. Some examples for such multispheres are displayed in Fig. 7

### Multispheres of multi-domain vesicles

Next, let us consider vesicle membranes with several *a*- and *b*-domains and, thus, with more than one domain boundary. All domains are taken to be in chemical equilibrium as described in the next subsection.

#### Chemical equilibrium between all domains

In order to distinguish the different *a*- and *b*-domains, we label them by the integers *k* and *n*, respectively. The membrane areas of the $a_k$- and $b_n$-domains are denoted by $A_{ak}$ and $A_{bn}$. In chemical equilibrium, the coexisting *a*- and *b*-phases are characterized by two different molecular compositions, where one composition applies to all *a*-domains and the other composition to all *b*-domains. In order to allow the domains to form different domain patterns, we introduce Lagrange multipliers $\Sigma _a$ and $\Sigma _b$, which are conjugate to the total surface area of all *a*-domains and to the total surface area of all *b*-domains, respectively. The corresponding shape functional $F_{> 2 \textrm{Do}}$ is obtained by generalizing the shape functional $F_{2 \textrm{Do}}$ for a two-domain vesicle as given by Eq. (A7) in Appendix A. Indeed, apart from the pressure term, each term of the shape functional $F_{2 \textrm{Do}}$ in Eq. (A7) is replaced by a sum over the different $a_k$- and $b_n$-domains. In particular, the two tension terms in Eq. (A7) are substituted according to [34]

70 $$\begin{aligned} \Sigma _a A_a + \Sigma _b A_b \rightarrow \Sigma _a \sum _k A_{ak} + \Sigma _b \sum _n A_{bn} \,. \end{aligned}$$

In general, each $a_k$-domain and each $b_n$-domain can now form a multisphere, in close analogy to the multispheres formed by uniform membranes as described in Sect. 2. When the $a_k$-domain forms a multisphere, the individual $a_{k}$-spheres are labeled by the index *i* and have the area $A_{aki}$ as well as the mean curvature $M_{aki}$. It then follows from the first variation of the generalized shape functional $F_{> 2 \textrm{Do}}$ that the mean curvature $M_{aki}$ satisfies the local shape equation

71 $$\begin{aligned} {\Delta \!P}= 2\, \Sigma ^\textrm{tot}_a M_{aki} - 4 \kappa _a m_a M_{aki}^2 \,, \end{aligned}$$

with the total membrane tension

72 $$\begin{aligned} \Sigma ^\textrm{tot}_a = \Sigma _a + 2 \kappa _a m_a^2 \end{aligned}$$

which is completely analogous to the local shape equation for uniform membranes as given by Eq. (1). One should note that the Lagrange multiplier $\Sigma _a$ and the curvature-elastic parameters $\kappa _a$ and $m_a$ are independent of the domain-index *k* and of the individual sphere index *i*.^2^

When the $b_n$-domain forms a multisphere, the individual $b_{n}$-spheres are labeled by the index *j*. The mean curvature $M_{bnj}$ of an individual $b_{nj}$-sphere formed by the $b_n$-domain fulfills the local shape equation

73 $$\begin{aligned} {\Delta \!P}= 2\, \Sigma ^\textrm{tot}_b M_{bnj} - 4 \kappa _b m_b M_{bnj}^2 \,, \end{aligned}$$

with the total membrane tension

74 $$\begin{aligned} \Sigma ^\textrm{tot}_b = \Sigma _b + 2 \kappa _b m_b^2 \end{aligned}$$

which is again completely analogous to Eq. (1).

**Fig. 8:**
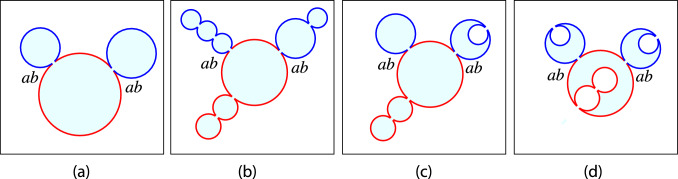
Some examples for multispherical shapes with two *ab*-necks that can be formed by vesicles with three domains in chemical equilibrium: **a** Three-sphere vesicles with one *a*-sphere (red) and two *b*-spheres (blue); **b**–**d** Multisphere vesicles with one cluster of three *a*-spheres (red) and two clusters of *b*-spheres (blue). Chemical equilibrium implies that, in each panel, the two *b*-clusters consist of large and small spheres with identical mean curvatures. Both *ab*-necks are positive in all four panels. In (**c**) and (**d**), the *bb*-necks are negative. In (**d**), the *aa*-necks are negative as well

The quadratic form of Eq. (71) for the mean curvature $M_{aki}$ implies that each $a_k$-domain forms $a_{ki}$-spheres with up to two different radii, provided by large $a_{ki}$-spheres with radius $R_{al}$ and by small $a_{ki}$-spheres with radius $R_{as}$. Likewise, the quadratic form of Eq. (73) for $M_{bnj}$ has the consequence that the $b_n$-domain forms $b_{nj}$-spheres with up to two different radii, $R_{bl}$ and $R_{bs}$, corresponding to large and small $b_{nj}$-spheres. Some examples for multispheres arising from three-domain vesicles in chemical equilibrium are displayed in Fig. 8. In these examples, the vesicle membrane consists of one *a*-domain and two *b*-domains, forming different clusters of *a*- and *b*-spheres, which are connected by two *ab*-necks.

#### Geometry of multispheres

As before, the individual *a*-spheres are labeled by $a_{ki}$, where the integer *k* labels the $a_k$-domain and the integer *i* the individual $a_{ki}$-spheres formed by the $a_k$-domain. Likewise, the individual *b*-spheres are labeled by $b_{nj}$ where the integer *n* is the index of the $b_n$-domain and the integer *j* labels a certain $b_{nj}$-sphere formed by the $b_n$-domain. The $a_{ki}$-sphere has the radius $R_{aki}$ and the rescaled radius $r_{aki} = R_{aki}/R_\textrm{ve}$; the $b_{nj}$-sphere has the radius $R_{bnj}$ and the rescaled radius $r_{bnj} = R_{bnj}/R_\textrm{ve}$.

The $a_k$-domain with area $A_{ak}$ and the $b_n$-domain with area $A_{bn}$ are now characterized by the area fractions

75 $$\begin{aligned} \Phi _{ak} = \frac{A_{ak}}{A} = \sum _i r_{aki}^2 \end{aligned}$$

and

76 $$\begin{aligned} \Phi _{bn} = \frac{A_{bn}}{A} = \sum _j r_{bnj}^2 \end{aligned}$$

with the total area fractions of the *a*- and *b*-domains as given by

77 $$\begin{aligned} \Phi _a = \sum _k \Phi _{ak} = \sum _k \sum _i r_{aki}^2 \end{aligned}$$

and

78 $$\begin{aligned} \Phi _b = \sum _n \Phi _{bn} = \sum _n \sum _j r_{bnj}^2 \,. \end{aligned}$$

Therefore, in contrast to the two-sphere vesicles in Sect. 3.3, all rescaled radii now fulfill the inequalities

79 $$\begin{aligned} r_{aki} \le \sqrt{\Phi _a} \quad \textrm{and} \quad r_{bnj} \le \sqrt{\Phi _b} \,, \end{aligned}$$

where the equality signs apply to a single *a*-domain forming a single *a*-sphere and to a single *b*-domain forming a single *b*-sphere, respectively.

Furthermore, the total volume enclosed by all *a*-spheres and all *b*-spheres is denoted by $V_a$ and $V_b$, respectively, which leads to the rescaled volumes

80 $$\begin{aligned} v_a \equiv \frac{V_a}{\frac{4\pi }{3} R_\textrm{ve}^3} \quad \textrm{and} \quad v_b \equiv \frac{V_b}{\frac{4\pi }{3} R_\textrm{ve}^3} \,. \end{aligned}$$

If all spheres have a positive mean curvature as in Figs. 7a, c and 8a, b, the rescaled volumes are given by

81 $$\begin{aligned} v_a = \sum _k \sum _i r_{aki}^3 \quad \textrm{and} \quad v_b = \sum _n \sum _j r_{bnj}^3 \,. \end{aligned}$$

If some *a*- or *b*-spheres have a negative mean curvature and enclose some part of the exterior compartment as in Figs. 7b, d and 8c, d, we have to substract the subvolumes of these spheres from the combined volume of the other spheres with a positive mean curvature.

#### Stability of *ab*-necks

To discuss the stability of an *ab*-neck between the $a_k$- and the $b_n$-domain, we label the *a*-sphere adjacent to the *ab*-neck by $a_{k1}$ and the *b*-sphere adjacent to the *ab*-neck by $b_{n1}$. The stability condition for a *positive* *ab*-neck is then given by:

82 $$\begin{aligned} \kappa _a \left( {\bar{m}}_a - \frac{1}{r_{ak1}} \right) + \kappa _b \left( {\bar{m}}_b - \frac{1}{r_{bn1}} \right) + \frac{\lambda R_\textrm{ve}}{2} \ge 0\nonumber \\ \end{aligned}$$

which has the same form as Eq. (46) but with the radii $r_a$ and $r_b$ replaced by the radii $r_{ak1} \le r_a$ and $r_{bn1} \le r_b$, leading to the mean curvatures $M_{ak1} = 1/r_{ak1} \ge 1/r_a = 1/ \sqrt{\Phi _a}$ and $M_{bn1} = 1/r_{bn1} \ge 1/r_b = 1/ \sqrt{\Phi _b}$, as follows from Eq. (79). The stability condition in Eq. (82) can be rewritten in the form

83 $$\begin{aligned} {\bar{m}}_b \ge h_\textrm{out}({\bar{m}}_a) \quad \textrm{or} \quad y \ge h_\textrm{out}(x) \end{aligned}$$

with $x = {\bar{m}}_a$ and $y = {\bar{m}}_b$ as before. The limiting case $y = h_\textrm{out}(x)$ describes the limit shapes $L_{ab}$ for a positive *ab*-neck. These limit shapes define a straight line in the (*x*, *y*)-plane, which is quite similar to the purple $L_{ab}$-line in Fig. 6a.

The stability condition for a *negative* *ab*-neck with an in-budded *b*-sphere has the form:

84 $$\begin{aligned} \kappa _a \left( {\bar{m}}_a - \frac{1}{r_{ak1}} \right) + \kappa _b \left( {\bar{m}}_b + \frac{1}{r_{bn1}} \right) - \frac{\lambda R_\textrm{ve}}{2} \le 0 \,,\nonumber \\ \end{aligned}$$

which has the same form as Eq. (56) but with the radii $r_a$ and $r_b$ replaced by the radii $r_{ak1} \le r_a$ and $r_{bn1} \le r_b$, leading to the mean curvatures $M_{ak1} = +1/r_{ak1} \ge 1/r_a$ and $M_{bnj} = -1/r_{bn1} \le - 1/r_a$. The stability condition in Eq. (84) can be rewritten in the form:

85 $$\begin{aligned} {\bar{m}}_b \ge h_{b\text {-in}}({\bar{m}}_a) \quad \textrm{or} \quad y \ge h_{b\text {-in}}(y) \,. \end{aligned}$$

The limiting case ${\bar{m}}_b = h_{b\text {-in}}({\bar{m}}_a)$ describes the limit shapes $L_{ab}$ for a negative *ab*-neck. These limit shapes are located along a straight line in the (*x*, *y*) plane, which is quite similar to the purple $L_{ab}$-line in Fig. 6b.

#### Stability of *aa*- and *bb*-necks

**Fig. 9:**
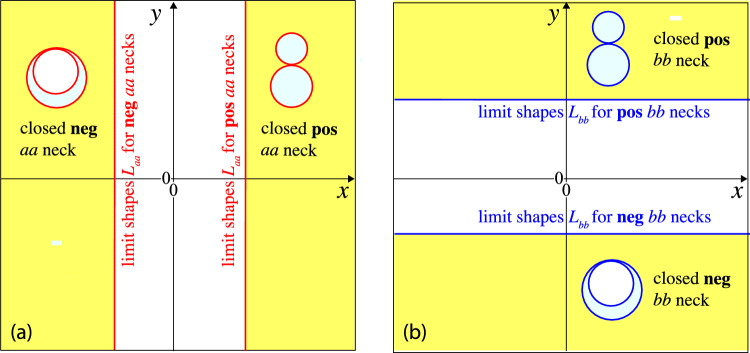
Morphology diagrams with coordinates $x = {\bar{m}}_a$ and $y= {\bar{m}}_b$: **a** Stability regimes (yellow) for positive and negative *aa*-necks. For positive *aa*-necks, the spontaneous curvature ${\bar{m}}_a \ge {\bar{M}}_{aa}^\textrm{eff}> 0$ as in Eq. (86), which defines the right stability regime. For negative *aa*-necks, the spontaneous curvature ${\bar{m}}_a \le {\bar{M}}_{aa}^\textrm{eff}< 0$ as in Eq. (87), leading to the left stability regime. Because the left and the right stability regime have no overlap, all *aa*-necks must be either positive or negative; and **b** Stability regimes (yellow) for positive and negative *bb*-necks. For positive *bb*-necks, the spontaneous curvature ${\bar{m}}_b \ge {\bar{M}}_{bb}^\textrm{eff}> 0$ as in Eq. (88), corresponding to the upper stability regime. For negative *bb*-necks, the spontaneous curvature ${\bar{m}}_a \le {\bar{M}}_{aa}^\textrm{eff}< 0$ as in Eq. (89), which defines the lower stability regime. Because the lower and the upper stability regime have no overlap, all *bb*-necks must be either positive or negative

In general, the multispheres consist of *ab*-necks as well as *bb*- and *aa*-necks, see the examples in Figs. 7 and  8. Each *ab*-neck can be positive or negative as described in Sect. . In addition, each *a*-domain forms one *a*-sphere or a cluster of several *a*-spheres connected by *aa*-necks. Likewise, each *b*-domain forms one *b*-sphere or a cluster of *b*-spheres connected by *bb*-necks. All *aa*-necks are either positive or negative and likewise for the *bb*-necks. Indeed, the stability regimes for positive and negative *aa*- or *bb*-necks have no overlap in the morphology diagrams defined by the two spontaneous curvatures $x = {\bar{m}}_a$ and $y={\bar{m}}_b$, see Fig. 9.

First, consider two *a*-spheres with rescaled radii $r_{ak1} = R_{ak1}/R_\textrm{ve}$ and $r_{ak2} = R_{ak2}/R_\textrm{ve}$, which are connected by a closed *aa*-neck. It follows from Eq. (17) that a positive *aa*-neck is stable if the rescaled curvatures fulfill the inequality

86 $$\begin{aligned} x = {\bar{m}}_a \ge {\bar{M}}_{aa}^\textrm{eff}= \frac{1}{2} \left( \frac{1}{r_{ak1}} + \frac{1}{r_{ak2}} \right) >0 \quad \text {(pos }aa\text {)} \,,\nonumber \\ \end{aligned}$$

which defines the right stability regime in Fig. 9a. On the other hand, a negative *aa*-neck with $r_{ak1} > r_{ak2}$ is stable provided

87 $$\begin{aligned} x = {\bar{m}}_a \le {\bar{M}}_{aa}^\textrm{eff}= \frac{1}{2} \left( \frac{1}{r_{ak1}} - \frac{1}{r_{ak2}} \right) <0 \quad \text {(neg }aa\text {)}\nonumber \\ \end{aligned}$$

as follows from Eq. (18), leading to the left stability regime in Fig. 9a.

Next, consider two *b*-spheres with rescaled radii $r_{bn1} = R_{bn1} /R_\textrm{ve}$ and $r_{bn2} = R_{bn2}/R_\textrm{ve}$, which are connected by a closed *bb*-neck. Equation (17) now implies that a positive *bb*-neck is stable if

88 $$\begin{aligned} y = {\bar{m}}_b \ge {\bar{M}}_{bb}^\textrm{eff}\equiv \frac{1}{2} \left( \frac{1}{r_{bn1}} + \frac{1}{r_{bn2}} \right) >0 \quad \text {(pos }bb\text {)} \,,\nonumber \\ \end{aligned}$$

corresponding to the upper stability regime in Fig. 9b. On the other hand, a negative *bb*-neck with $r_{bn1} > r_{bn2}$ is stable for

89 $$\begin{aligned} y = {\bar{m}}_b \le {\bar{M}}_{bb}^\textrm{eff}\equiv \frac{1}{2} \left( \frac{1}{r_{bn1}} - \frac{1}{r_{bn2}} \right) <0 \quad \text {(neg }bb\text {)}\nonumber \\ \end{aligned}$$

as in Eq. (18), which defines the lower stability regime in Fig. 9b.

## Multispherical shapes of two-domain vesicles

In this section, the stability of multispherical shapes formed by vesicles with one *a*- and one *b*-domain will be examined in more detail. These multispheres involve both a single *ab*-neck and additional *aa*- and *bb*-necks between two *a*-spheres and two *b*-spheres. The stability of each neck is governed by its own stability condition as described in Sects. 5.2.3 and 5.2.4. In order to identify the parameter regimes of stable multispheres, we need to impose and combine the stability conditions for all membrane necks, which are present in the multisphere.

### Four-sphere shapes with positive *ab*-neck

**Fig. 10:**
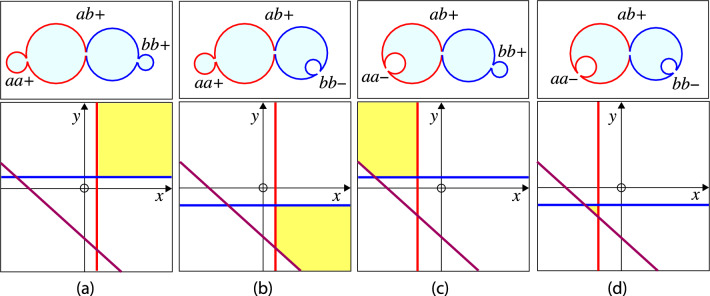
Four-sphere shapes and their stability regimes for *positive* *ab*-necks. The multispheres consist of one *a*-domain (red) and one *b*-domain (blue) forming two *a*-spheres and two *b*-spheres: **a** Positive *aa*-neck and positive *bb*-neck; **b** positive *aa*-neck and negative *bb*-neck; **c** negative *aa*-neck and positive *bb*-neck; and **d** Negative *aa*-neck and negative *bb*-neck. The bottom row displays the stability regimes (yellow) within the morphology diagrams defined by the rescaled spontaneous curvatures $x= {\bar{m}}_a$ and $y = {\bar{m}}_b$. The purple, red, and blue lines represent the lines of limit shapes $L_{ab}$, $L_{aa}$, and $L_{bb}$, respectively, which provide the boundaries for the stability regimes. In (**d**), the three lines lead to a small, triangular stability regime, which requires fine-tuning of both spontaneous curvatures

Representative examples for four-sphere shapes with one positive *ab*-neck are displayed in Fig. 10. These multispheres consist of two *a*-spheres connected by a single *aa*-neck and of two *b*-spheres connected by a single *bb*-neck. Each of the four spheres can have a different mean curvature, in accordance with the shape equations for the *a*- and *b*-spheres. Both the *aa*-neck and the *bb*-neck can be positive or negative, which implies four different types of four-sphere shapes with positive *ab*-necks as in Fig. 10. In each panel of this figure, the top and bottom subpanels display one of the four-sphere shapes together with the corresponding stability regime within the morphology diagram defined by the rescaled spontaneous curvatures $x = {\bar{m}}_a$ and $y = {\bar{m}}_b$.

Each four-sphere shape in the upper row of Fig. 10 involves three closed necks, each of which is governed by a different stability condition. These three stability conditions determine three half-planes, which represent the stability regimes for the three individual necks. The intersection of these three half-planes determines the stability regime of the four-sphere shape under consideration. Each half-plane is bounded by a line of limit shapes, denoted by $L_{ab}$, $L_{aa}$, and $L_{bb}$. Thus, the lower subpanels of Fig. 10 display three lines of limit shapes, purple $L_{ab}$-lines as in Fig. 6a as well as red $L_{aa}$-lines and blue $L_{bb}$-lines as in Fig. 9.

In Fig. 10a, all four spheres have positive mean curvatures which implies that all three necks are positive. Four-sphere shapes with stable *ab*-necks must be located to the right of the purple $L_{ab}$-line as in Fig. 6a. Furthermore, the positive *aa*-neck confines the stability regime of the four-sphere shape to positive values of $x = {\bar{m}}_a$ as in Fig. 9a, and the positive *bb*-neck is only stable for sufficiently positive values of $y = {\bar{m}}_b$ as in Fig. 9b. As a consequence, the yellow stability regime in the bottom row of Fig. 10a is confined to the upper right quadrant of the (*x*, *y*)-plane, which implies that the stability of the four-sphere shape in Fig. 10a requires sufficiently large spontaneous curvatures ${\bar{m}}_a = x$ and ${\bar{m}}_b = y$.

In Fig. 10b, the *bb*-neck is negative which moves the yellow stability regime to negative values of $y = {\bar{m}}_b$ and thus to the lower right quadrant of the morphology diagram On the other hand, Fig. 10c involves a negative *aa*-neck, which moves the stability regime to negative values of $x = {\bar{m}}_a$ and thus to the upper left quadrant of the morphology diagram. Finally, the four-sphere shape in Fig. 10d involves both a negative *aa*- and a negative *bb*-neck. In the latter case, the stability regime is confined to the small triangle formed by the three lines of limit shapes. Therefore, the formation of the multisphere in Fig. 10d requires fine-tuning of the two spontaneous curvatures ${\bar{m}}_a$ and ${\bar{m}}_b$.

### Four-sphere shapes with negative *ab*-neck

**Fig. 11:**
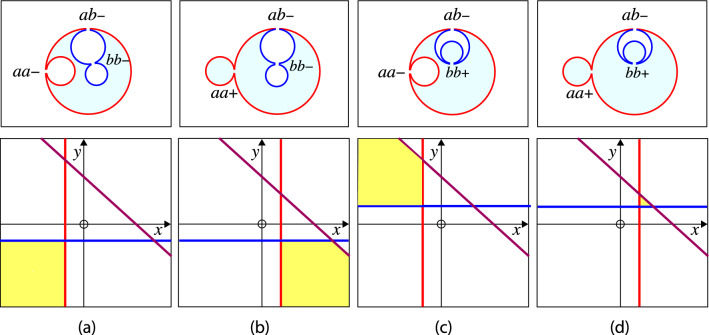
Four-sphere shapes and their stability regimes for *negative* *ab*-necks. The multispheres consist of one *a*-domain (red) and one in-budded *b*-domain (blue), forming two *a*-spheres and two *b*-spheres: **a** Negative *aa*-neck and negative *bb*-neck; **b** Positive *aa*-neck and negative *bb*-neck; **c** Negative *aa*-neck and positive *bb*-neck; and **d** Positive *aa*-neck and positive *bb*-neck. The subpanels in the bottom row display the corresponding stability regimes (yellow) within the morphology diagram defined by the rescaled spontaneous curvatures $x= {\bar{m}}_a$ and $y = {\bar{m}}_b$. The purple, red, and blue lines represent the lines of limit shapes $L_{ab}$, $L_{aa}$, and $L_{bb}$, respectively, which provide the boundaries for the stability regimes of the four-sphere shapes. In (**d**), the three lines lead to a small, triangular stability regime, which requires fine-tuning of both spontaneous curvatures

Four-sphere shapes with one negative *ab*-neck are displayed in Fig. 11. In these examples, the negative *ab*-neck arises from the in-budded *b*-domain. Each four-sphere shape consists of two *a*-spheres and two *b*-spheres. Each of these four spheres can have a different radius, in accordance with the two shape equations for the *a*- and *b*-domain. In addition to the negative *ab*-neck, the multispheres in Fig. 11 again involve a single *aa*-neck and a single *bb*-neck, both of which can be positive or negative, generating four different four-sphere shapes with a negative *ab*-neck. In each panel of Fig. 11, the top and bottom subpanels display one of these four-sphere shapes and the corresponding stability regime within the morphology diagram as defined by the rescaled spontaneous curvatures $x = {\bar{m}}_a$ and $y = {\bar{m}}_b$.

All four-sphere shapes in the upper row of Fig. 11 involve one *aa*-neck and one *bb*-neck in addition to the negative *ab*-neck. Each neck is stably closed when it fulfills the associated stability condition. Each of these conditions again defines a half-plane in the morphology diagram. The intersection of these three half-planes determines the stability regime of the four-sphere shape. Furthermore, each half-plane in Fig. 11 is bounded by a line of limit shapes as displayed in the lower subpanels of Fig. 11: purple $L_{ab}$-lines as in Fig. 6b as well as red $L_{aa}$-lines and blue $L_{bb}$-lines as in Fig. 9.

In Fig. 11a, the *a*-domain forms an in-bud which implies a negative *aa*-neck. Furthermore, the in-budded *b*-domain consists of two *b*-spheres with negative mean curvature, which leads to a negative *bb*-neck. The negative *aa*-neck and the negative *bb*-neck are stably closed for sufficiently large negative values of the spontaneous curvatures $x = {\bar{m}}_a$ and $y= {\bar{m}}_b$. Therefore, the yellow stability regime of this shape is confined to the lower left quadrant of the (*x*, *y*)-plane.

In Fig. 11b, the *aa*-neck is positive whereas the *bb*-neck is negative. The positive *aa*-neck shifts the stability regime to positive values of $x = {\bar{m}}_a$ and thus to the lower right quadrant of the (*x*, *y*)-plane. In Fig. 11c, the *aa*-neck is negative whereas the *bb*-neck is positive. The positive *bb*-neck shifts the stability regime to positive values of $y = {\bar{m}}_b$ and thus to the upper left quadrant of the (*x*, *y*)-plane. Finally, the four-sphere shape in Fig. 11d involves both a positive *aa*- and a positive *bb*-neck. In the latter case, the stability regime is confined to the small triangle enclosed by the three lines of limit shapes. Therefore, the formation of the shape displayed in Fig. 11d requires fine-tuning of the two spontaneous curvatures ${\bar{m}}_a$ and ${\bar{m}}_b$.

### Multispheres with multiple *aa*- and *bb*-necks

In general, a two-domain vesicle can form multispheres that consist of an *a*-cluster with more than two *a*-spheres and a *b*-cluster with more than two *b*-spheres. The *a*-cluster is built up from large *a*-spheres with radius $R_{al}$ and small spheres with radius $R_{as}$ as follows from the local shape equation in Eq. (71). Furthermore, Sect.  and  for uniform membranes imply that all *aa*-necks are either positive or negative. Thus, the *a*-cluster can attain two global architectures, corresponding to cases I and II for uniform membranes.

For case I, the large and small *a*-spheres have positive mean curvature and are connected by positive *aa*-necks. For case II, the *a*-cluster is provided by one large *a*-sphere with positive mean curvature and multiple small *a*-spheres with negative mean curvature, with all *a*-spheres being connected by negative *aa*-necks. The same two cases can be distinguished for the *b*-cluster. For case I, the large and small *b*-spheres have positive mean curvature and are connected by positive *bb*-necks. For case II, the *b*-cluster consists of one large *b*-sphere with positive mean curvature and one or several small *b*-spheres with negative mean curvature, with all *b*-spheres being connected by negative *bb*-necks.

In general, both the *a*- and the *b*-clusters can involve different types of necks: *ss*-necks between two small spheres; *ls*-necks between a large and a small sphere; and *ll*-necks between two large spheres. The *a*- and *b*-cluster of the seven-sphere shapes in Fig. 7c, d, for example, involve both *ls*-necks and *ss*-necks. The stability of the multisphere is then determined by the least stable necks which impose the strongest closed neck condition on the spontaneous curvatures.

If the cluster of *a*-spheres belongs to case I with positive mean curvatures of the large and small *a*-spheres, the cluster consists, in general, of large and small spheres, which can be connected by *ss*-, *ls*-, or *ll*-necks. The effective mean curvatures of these necks are ordered according to

90 $$\begin{aligned} {\bar{M}}_{ss}^\textrm{eff}> {\bar{M}}_{ls}^\textrm{eff}> {\bar{M}}_{ll}^\textrm{eff}> 0 \,. \end{aligned}$$

Therefore, all necks of the *a*-cluster are stable for sufficiently large and positive spontaneous curvature

91 $$\begin{aligned} {\bar{m}}_a \ge M_{ss}^\textrm{eff}> M_{ls}^\textrm{eff}> M_{ll}^\textrm{eff}\quad \text {(Case I).} \end{aligned}$$

A special case I is obtained if all spheres of the *a*-cluster have the same rescaled radius $$r_{a*}$$. Such a multisphere consisting of equally sized *a*-spheres has the smallest rescaled volume $$v_a$$ of all multispheres with the same total number of *a*-spheres [25, 26]. In the latter case, all necks have the same effective mean curvature $${\bar{M}}_{**}^\textrm{eff}= 1/r_{a*}$$. These necks are stable if the spontaneous curvature is large and positive with

92 $$\begin{aligned} {\bar{m}}_a \ge {\bar{M}}_{**}^\textrm{eff}\quad \text {(Case I, equally sized spheres).} \end{aligned}$$

One example for a multisphere consisting of equally sized spheres as formed by a uniform membrane is displayed in Fig. 2g.

If the cluster of *a*-spheres belongs to case II, it consists of one large *a*-sphere with positive mean curvature and one or several small *a*-spheres with negative mean curvature. Such an *a*-cluster involves only *ls*- and *ss*-necks with negative neck curvatures $M_{ls}^\textrm{eff}< 0$ and $M_{ss}^\textrm{eff}< 0$. These necks are stable if

93 $$\begin{aligned} {\bar{m}}_a \le M_{ss}^\textrm{eff}< M_{ls}^\textrm{eff}< 0 \quad \text {(Case II).} \end{aligned}$$

The neck stability of the *b*-cluster is obtained by replacing the domain label *a* in Eqs. (91), (92), and (93) by the domain label *b*. It follows from these stability conditions for the *a*- and *b*-cluster that the qualitative features of the morphology diagrams as shown in Figs. 10 and 11 for four-sphere shapes also apply to two-domain vesicles with more than two *a*-spheres and/or more than two *b*-spheres.

## Nested multispheres from nested domains

**Fig. 12:**
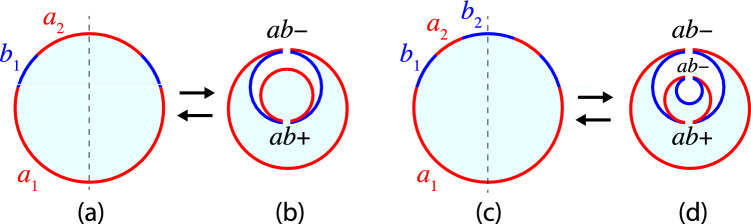
Trasformation of nested multi-domain vesicles into nested multispheres: **a** a spherical three-domain vesicle consisting of a large $a_1$-domain (red) on the southern hemisphere, a smaller ring-like $b_1$-domain (blue) on the northern hemisphere, and an even smaller $a_2$-domain (red) close to the north pole. Deflation of such a three-domain vesicle can lead to the nested multisphere in (**b**) with an outer $a_1$-sphere, an in-budded $b_1$-sphere, and an out-budded $a_2$-sphere; and **c** a spherical four-domain vesicle consisting of a large $a_1$-domain (red), a ring-like $b_1$-domain (blue), a ring-like $a_2$-domain (red), and a $b_2$-domain (blue) close to the north pole. For simplicity, the three- and four-domain vesicles in (**a**) and (**c**) are taken to be axisymmetric, which implies that the nested domains are concentric as well. However, shifting the concentric domains against each other will again lead to nested multispheres, which are quite similar to those in (**b**) and (**d**)

A special case of multispheres with several *ab*-necks is obtained starting from nested domains. For a spherical vesicle, the simplest example for such a domain pattern is displayed by the three-domain vesicle in Fig. 12a. In this example, the southern hemisphere of the vesicle together with a small fraction of the northern hemisphere is covered by a large $a_1$-domain, while the northern hemisphere contains a ring-like $b_1$-domain, which encloses an even smaller $a_2$-domain at the north pole. Note that the $a_1$- and the $a_2$-domain have the same molecular composition and thus possess the same spontaneous curvature ${\bar{m}}_a$. Likewise, the $b_1$- and the $b_2$-domain in Fig. 12c are characterized by the same spontaneous curvature ${\bar{m}}_b$. Furthermore, all domain boundaries have the same line tension $\lambda$.

### Nested multispheres with two *ab*-necks

Deflation of the vesicle in Fig. 12a can lead to the nested multisphere displayed in Fig. 12b. The latter multisphere consists of three spheres that are nested into each other. The $a_1$-domain forms the largest sphere with radius $R_{a1}$ and positive mean curvature $M_{a1} = + 1/R_{a1} >0$, whereas the ring-like $b_1$-domain has transformed into the $b_1$-sphere with radius $R_{b1}$ and negative mean curvature $M_{b1} = - 1/R_{b1} < 0$. In addition, the small $a_2$-domain, which was located close to the north pole of the spherical vesicle in Fig. 12a, now forms the smallest sphere with radius $R_{a2}$ and positive mean curvature $M_{a2} = + 1/R_{a2} > 0$. The requirement that the membrane of the nested multisphere in Fig. 12b should not intersect itself implies that the three spherical radii must satisfy the inequalities $R_{a1}> R_{b1} > R_{a2}$ and that the areas of the three domains are thus ordered according to $A_{a1}> A_{b1} > A_{a2}$.

The nested multisphere in Fig. 12b involves one negative and one positive *ab*-neck. The negative *ab*-neck connects the outer $a_1$-sphere with the in-budded $b_1$-sphere. The corresponding stability condition is described by Eq. (84) and leads to a line of limit shapes $L_{ab}$ as displayed in Fig. 6b. The negative *ab*-neck is stable for *x*- and *y*-values *below* the $L_{ab}$-lines in Fig. 6b, with the coordinates *x* and *y* of the morphology diagram provided by the spontaneous curvatures ${\bar{m}}_a$ and ${\bar{m}}_b$, respectively. The positive *ab*-neck of the nested multisphere in Fig. 12b is located between the in-budded $b_1$-sphere and the out-budded $a_2$-sphere. This positive *ab*-neck is stable for *x*- and *y*-values *above* the $L_{ab}$-line in Fig. 6a. As previously discussed in Sect. 3.5.3, the stability regimes for negative and positive *ab*-necks exhibit a large overlap region in the morphology diagram. The nested multisphere in Fig. 12b is stable for values of the spontaneous curvatures ${\bar{m}}_a$ and ${\bar{m}}_b$ within this overlap region.

The formation of the $$a_1$$- and $$a_2$$-spheres with positive mean curvatures $$M_{a1} >0 $$ and $$M_{a2}>0$$ will be facilitated by positive spontaneous curvature $$x = {\bar{m}}_a >0$$. Likewise, the formation of the $$b_1$$-sphere with negative mean curvature $$M_{b1} < 0$$ will be supported by negative spontaneous curvature $$y = {\bar{m}}_b < 0$$. However, the absolute values of $${\bar{m}}_a$$ and $${\bar{m}}_b$$ must be sufficiently small so that the two spontaneous curvatures $$x = {\bar{m}}_a$$ and $$y = {\bar{m}}_b$$ define a point (*x*, *y*) of the morphology diagram that is located within the overlap region of the yellow stability regimes in Fig. 6a, b.

### Nested multispheres with three *ab*-necks

Another example for a nested domain pattern is displayed by the four-domain vesicle in Fig. 12c. This domain pattern involves the same $a_1$- and $b_1$-domains as the pattern in Fig. 12a but the $a_2$-domain now forms another ring-like domain, enclosing a fourth $b_2$-domain at the north pole. Deflation of such a vesicle can lead to four nested spheres as displayed in Fig. 12d. The $a_1$-domain again forms the largest sphere with radius $R_{a1}$ and positive mean curvature $M_{a1} = + 1/R_{a1} >0$, whereas the ring-like $b_1$-domain again forms the $b_1$-sphere with radius $R_{b1}$ and negative mean curvature $M_{b1} = - 1/R_{b1} < 0$. Furthermore, the ring-like $a_2$-domain now turns into the $a_2$-sphere with positive mean curvature $M_{a2} = + 1/R_{a2} > 0$. Finally, the $b_2$-domain close to the north pole becomes the in-budded $b_2$-sphere with negative mean curvature $M_{b2} = - 1/R_{b2} < 0$. The requirement that the membrane of the nested multisphere in Fig. 12d should not intersect itself implies that the four spherical radii satisfy the inequalities $R_{a1}> R_{b1}> R_{a2} > R_{b2}$.

The nested multisphere in Fig. 12d involves two negative and one positive *ab*-necks. One negative *ab*-neck connects the outer $a_1$-sphere with the in-budded $b_1$-sphere. The positive *ab*-neck is located between the in-budded $b_1$-sphere and the out-budded $a_2$-sphere. The stability regimes for these two *ab*-necks are very similar to the stability regimes for the two *ab*-necks in Fig. 12b. In addition, the nested multisphere in Fig. 12d contains another negative *ab*-neck, which connects the out-budded $a_2$-sphere with the in-budded $b_2$-sphere.

The nested multisphere displayed in Fig. 12d involves two *a*-spheres with positive mean curvatures $M_{a1}>0$ and $M_{a2} > M_{a1}$ and two *b*-spheres with negative mean curvatures $M_{b1}< 0$ and $M_{b2} < M_{b1}$. We could try to add another level of nesting, by adding an $a_3$-domain within the $b_2$-domain close to the north pole. Such a domain pattern is possible but cannot lead, in chemical equilibrium, to a nested multisphere because such a multisphere would involve three *a*-spheres with three different mean curvatures, which is inconsistent with the local shape equation as given by Eq. (71). On the other hand, we can connect another *a*-sphere with positive mean curvature $M_{a2}$ to the existing $a_1$-sphere, thereby creating a positive *aa*-neck or another *b*-sphere with negative mean curvature $M_{b2}$ to the existing $b_1$-sphere via a negative *bb*-neck. These additional *aa*- or *bb*-necks will introduce $L_{aa}$- or $L_{bb}$-lines as in Fig. 9, which further restrict the stability regimes as discussed in Sect. 5.2.4.

## Constant-mean-curvature (CMC) surfaces

### Conventional CMC surfaces

**Fig. 13:**
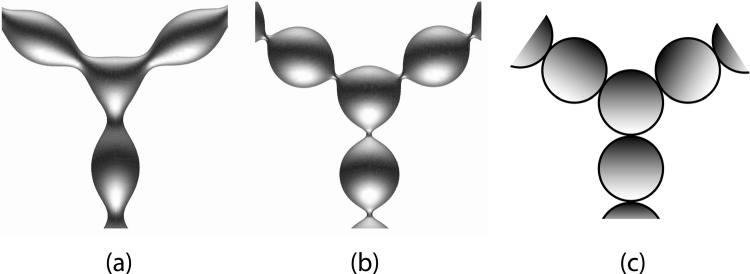
Multispherical limit of triunduloids: **a**, **b** Triunduloids with open membrane necks as studied in differential geometry. Reprinted with permission from Ref.  [40] (Copyright 1997, Springer-Verlag); and **c** Triunduloid built up from punctured spheres which are connected by closed necks. The central sphere is connected to three adjacent spheres by three necks

In the differential geometry of surfaces [35], multispherical shapes consisting of equally-sized spheres have been studied in the context of constant-mean-curvature (CMC) surfaces, generalizing the concept of minimal surfaces with zero mean curvature $M = 0$. For a long time, the only examples for freely suspended CMC surfaces with $M \ne 0$ were provided by the unduloids of Delaunay [36], which provide a one-parameter family of tubular shapes that interpolate smoothly between multispherical tubes consisting of equally sized (and punctured) spheres and cylindrical tubes. More recently, additional CMC surfaces have been constructed by pertubing a cluster of identical spheres that touch each other [37–41]. One example are triunduloids [40, 41] that consist of three unduloidal arms connected by a central core as displayed in Fig. 13.

The physical system typically used to motivate CMC surfaces are the shapes of soap films and liquid droplets. However, when the initial cluster of identical and touching spheres is viewed as a cluster of liquid droplets, the resulting CMC surface is not stable. Indeed, the cluster will either fall apart and then form many small droplets or it will coalesce into one large droplet that will eventually attain the shape of a single sphere. However, when the cluster of droplets is enclosed by a membrane, this membrane can lead to stable multispherical shapes as described in Sects. 3–7.

### Multispheres as generalized CMC surfaces

The conventional CMC surfaces considered in differential geometry have a constant mean curvature that is uniform along the whole surface. For the vesicle surfaces as considered here, examples for such conventional CMC surfaces are provided by multispheres consisting of equally sized spheres as in Fig. 2g. The latter vesicles are bounded by uniform membranes which have a laterally uniform composition as well as laterally uniform elastic properties. However, even uniform membranes can form multispheres with two different piece-wise constant mean curvatures as displayed in most panels of Fig. 2. Such multispheres with different piece-wise constant mean curvature should be regarded as generalized CMC surfaces.

As discussed in the previous sections, vesicles with intramembrane domains can form multispheres with up to four different piece-wise constant mean curvatures. Therefore, these multispheres provide new examples of generalized CMC surfaces. If we considered molecular membrane compositions that lead to the coexistence of three phases, we could obtain multispheres with up to six different piece-wise constant mean curvatures. In general, if the molecular composition can generate *N* coexisting phases, the multispherical shapes could involve up to 2*N* different values of the sphere radius.

## Experiments on multispherical vesicles

To experimentally study the multispherical shapes of multi-domain vesicles as determined here theoretically, it will be useful to combine and extend several experimental protocols. The first protocol corresponds to the same procedure as recently used for uniform membranes [25], see Fig. 2, but now applied to ternary lipid mixtures that undergo phase separation into two fluid phases. The second protocol, also developed quite recently [33], allows to control and fine-tune the membrane’s spontaneous curvature by the binding of His-tagged fluorophores to anchor lipids in the membranes. The third protocol, which has been introduced already some time ago [7, 9], generates multi-domain vesicles by electrofusion of membranes.

### Deflation combined with solution asymmetry

The multispheres displayed in Fig. 2 have been obtained for giant vesicles by a combination of osmotic deflation and solution asymmetry between the interior and exterior compartments of the vesicles, which were first prepared in a symmetric sucrose solution. Subsequently, a small aliquot of the prepared sucrose-vesicle solution was transferred into the observation chamber where they were added to a larger aqueous droplet that contained primarily glucose, with a glucose concentration that exceeded the sucrose concentration in the aliquot. This transfer or dilution step led to the reduction of the vesicle volume by fast osmotic deflation and, at the same time, to the generation of bilayer asymmetry and spontaneous curvature.

The lipid bilayers of the giant vesicles in Fig. 2 contained binary mixtures of the phospholipid POPC and cholesterol. In order to obtain a lipid bilayer that forms two coexisting fluid phases, a third lipid component such as another phospholipid or sphingomyelin should be added. Thus, the simplest experimental approach to generate multispheres with two or more intramembrane domains will be obtained when the experimental protocol developed in [25] is applied to such ternary lipid mixtures.

For the binary mixtures of POPC and cholesterol, the spontaneous curvatures generated by the sugar asymmetry between sucrose and glucose within the uniform bilayer membranes was of the order of $1/\mu$m.

### Fine-tuning of spontaneous curvatures

In order to fine-tune the spontaneous curvatures $m_a$ and $m_b$ of the *a*- and *b*-domains, it will be useful to dope the ternary lipid mixtures with some anchor lipids that bind His-tagged fluorophores from the exterior compartment. Such a method has been successfully applied to ternary lipid mixtures of POPC, POPG, and cholesterol exposed to His-tagged GFP [33]. In general, the density of the membrane-bound His-tagged fluorophores depends on the density of the anchor lipids as well as on the solution concentration. In Ref.  [33], rather large spontaneous curvatures of the order of 1/(100 nm) have been achieved by exposing the giant vesicles to nanomolar concentrations of His-tagged GFP.

As explained in Sects. 3 and 5 and illustrated in Figs. 6, 10, and 11, the morphology of the theoretically predicted multispheres depends strongly on the rescaled spontaneous curvatures $x = {\bar{m}}_a$ and $y = {\bar{m}}_b$ of the *a*- and *b*-domains. In general, the partitioning of the anchor lipids is expected to lead to different anchor lipid densities within the two types of domains. In addition, one might use two types of anchor lipids, each of which becomes enriched in one of the two membrane domains.

As a result, one should be able to obtain significantly different densities of membrane-bound fluorophores in the *a*- and *b*-domains and, thus, significantly different spontaneous curvatures ${\bar{m}}_a$ and ${\bar{m}}_b$. The bilayer asymmetries can be further enhanced by asymmetric sugar or ion solutions. In this way, it will become possible to explore large parameter regions of the morphology diagrams in Figs. 6, 10, and 11.

To experimentally obtain nested multispherical shapes as displayed in Fig. 12 will be particularly challenging. First, the *a* and *b* domains need to possess fine-tuned values of the spontaneous curvatures $x={\bar{m}}_a$ and $y={\bar{m}}_b$, which belong to the overlap region of the stability regimes in Figs. 6a and b. Second, to create such nested multispheres from a multidomain vesicle with a spherical shape, one should start from nested *a*- and *b*-domains as displayed in Fig. 12a, c.

### Multi-domain vesicles via electrofusion

In principle, one can create multi-domain vesicles by fusing two GUVs that are aspirated by two micropipettes. When the compositions of the two GUVs correspond to coexisting *a*- and *b*-phases, the fused GUVs should be composed of stable *a*- and *b*-domains. Fusion of two aspirated GUVs is, however, difficult to achieve because the aspirated vesicles tend to rupture. A more robust method is provided by electrofusion which has indeed been used to create multi-domain vesicles [7, 9].

In these studies, two populations of GUVs have been prepared from different lipid compositions. In Ref. [9], for example, the membranes of one vesicle population consisted of DOPC and CHOL whereas the membranes of the other population were composed of SM and CHOL. Electrofusing a vesicle from the DOPC/CHOL population with a vesicle from the SM/CHOL population then leads to a GUV membrane that contains all three lipid components.

The standard protocol for electrofusion of GUVs is similar to the electrofusion of cells [42, 43]. This protocol consists of two steps. First, the vesicles are aligned by an alternating electrical field, which brings them into close contact at their poles. Second, a short pulse of a high electric field is applied to the aligned vesicles, thereby electroporating the two vesicle membranes within their contact area.

## Different Gaussian curvature moduli

So far, the Gaussian curvature moduli $\kappa _{Ga}$ and $\kappa _{Gb}$ of the *a*- and *b*-domains were taken to have the same value. As mentioned in the introduction, it then follows that the domain boundaries are located within the closed *ab*-necks. At the end, let us look at the changes arising from different Gaussian curvature moduli, $\kappa _{Ga} \ne \kappa _{Gb}$ in the two domains. In this case, the matching condition in Eq. (40) implies that the domain boundary moves out of the waist-line of the closing neck. The closed neck is then formed by the membrane domain with the larger $\kappa _G$-value. Indeed, the Gaussian curvature *G* is negative around the closed neck and makes a more negative contribution to the Gaussian curvature energy in Eq. (A4) when this neck is formed by the domain with the larger $\kappa _G$-value.^3^ This conclusion also applies to negative values of the Gaussian curvatures moduli, that is, to $\kappa _{Ga} < 0$ and $\kappa _{Gb} < 0$..

### Shift of domain boundary

A simple estimate for the displacement of the domain boundary away from the closed neck can be obtained as follows. Such a displacement leads to the change $\Delta E_G$ in the Gaussian curvature energy as given by Eq. (A5). The largest possible energy gain arising from $\Delta E_G$ is given by

94 $$\begin{aligned} \min [\Delta E_G] = - 2 \pi | \kappa _{Ga} - \kappa _{Gb} |\,. \end{aligned}$$

This energy gain must overcompensate the line energy $\Delta E_\lambda$ of the domain boundary with radius $R_\textrm{db}$, which is equal to

95 $$\begin{aligned} \Delta E_\lambda = 2 \pi R_\textrm{db}\lambda \end{aligned}$$

with positive line tension $\lambda$. Therefore, the radius $R_\textrm{db}$ of the domain boundary satisfies the inequality

96 $$\begin{aligned} R_\textrm{db}< \frac{| \kappa _{Ga} - \kappa _{Gb} |}{\lambda } \,. \end{aligned}$$

For a uniform membrane, the Gaussian curvature modulus $\kappa _G$ is expected to be negative with a magnitude that is comparable to the bending rigidity $\kappa$ [44–46]. Therefore, the term $| \kappa _{Ga} - \kappa _{Gb} |$ should be comparable to $|\kappa _a - \kappa _b|$, which is of the order of $10^{-19}\,$J. Based on experimental studies of GUVs, the line tension $\lambda$ was estimated to lie within the range 1 pN and 0.01 pN, depending on the lipid composition of the GUV membranes [4, 6, 8]. It then follows from Eq. (96) that the radius $R_\textrm{db}$ of the domain boundary satisfies $R_\textrm{db}< 100\,$nm for $\lambda = 1\,$pN and $R_\textrm{db}< 1\,\mu$m for $\lambda = 0.1\,$pN. Therefore, the displacement of the domain boundary away from the closed membrane neck will not be detectable by conventional fluorescence microscopy if the line tension is of the order of 1 pN but should become visible for line tension values below 0.1 pN, in accordance with experimental observations [6, 8].

### Reduction of constriction forces

Now, assume that the *a*-domain represents the Ld lipid phase and the *b*-domain the Lo lipid phase, compare Fig. 1. The Lo phase is more rigid than the Ld phase which implies that the bending rigidity $\kappa _b$ of the *b*-domain exceeds the bending rigidity $\kappa _a$ of the *a*-domain, that is $\kappa _b > \kappa _a$. Using the previously mentioned estimates $\kappa _{G a} \simeq - \kappa _a$ and $\kappa _{G b} \simeq - \kappa _b$, the inequality $\kappa _a < \kappa _b$ implies that $\kappa _{G a} > \kappa _{G b}$ and that the neck is formed by the *a*-domain, that is, by the more flexible domain with the lower bending rigidity.

When the domain boundary moves out of the neck for $\kappa _{G a} > \kappa _{G b}$, the closed neck is located within the *a*-domain. More precisely, this neck provides a connection between the complete *a*-sphere and the narrow *a*-strip between the neck and the *b*-domain. The effective mean curvature of this neck is given by

97 $$\begin{aligned} M^\textrm{eff}_{a | a} \equiv \frac{1}{2} \left( M_{a-\textrm{sp}} + M_{a-\textrm{st}} \right) \end{aligned}$$

where $M_{a-\textrm{sp}}$ is the mean curvature of the complete *a*-sphere and $M_{a-\textrm{st}}$ is the mean curvature of the narrow *a*-strip on the other side of the neck.

A positive neck with neck curvature $M^\textrm{eff}_{a | a} > 0$ then experiences the constriction force

98 $$\begin{aligned} f = 8 \pi \kappa _a (m_a - M^\textrm{eff}_{a | a}) \quad \hbox { for}\ m_a \ge M^\textrm{eff}_{a | a} > 0 \end{aligned}$$

as follows from Eq. (64) with $\lambda = 0$ and $m_b = m_a$. This constriction force has the same form as for the positive neck of a uniform GUV membrane with bending rigidity $\kappa _a$ and spontaneous curvature $m_a$ [33].

On the other hand, a negative neck with neck curvature $M^\textrm{eff}_{a | a} < 0$ is subject to the constriction force

99 $$\begin{aligned} f = 8 \pi \kappa _a (M^\textrm{eff}_{a | a} - m_a) \quad \hbox { for}\ m_a \le M^\textrm{eff}_{a | a} < 0 \end{aligned}$$

as follows from Eq. (67) with $\lambda = 0$ and $m_b = m_a$. The constriction force as given by Eq. (99) has the same form as for the negative neck of a uniform GUV membrane with bending rigidity $\kappa _a$ and spontaneous curvature $m_a$ [47].

Therefore, if the *ab* domain boundary moves out of the neck during neck closure, the constriction force *f* as given by Eq. (98) for positive necks and by Eq. (99) for negative necks contains no contribution from the line tension $\lambda$, in contrast to Eqs. (64) and (67), which contain the term $2 \pi \lambda$ for both positive and negative *ab*-necks. Because the line tension is necessarily positive, the constriction forces as given by Eqs. (98) and (99) are reduced compared to the constriction forces in Eqs. (64) and (67). Nevertheless, the constriction forces in Eqs. (98) and  (99), which have the same form as the forces experienced by the closed necks of a uniform GUV membrane, can be sufficiently large to induce neck fission as demonstrated experimentally in Ref. [33].

## Conclusion

In this paper, multispherical shapes of vesicles were studied using the theory of curvature elasticity. We started with a brief review of multispheres formed by uniform membranes and introduced the distinction between positive and negative membrane necks based on the sign of the necks’ effective mean curvature (Sect. ). We then described multispheres formed by vesicles with two intramembrane domains, one *a*- and one *b*-domain, which arise from membrane phase separation into two fluid phases. These two-domain vesicles can form two-sphere shapes consisting of one *a*- and one *b*-sphere, connected by a single closed *ab*-neck.

Depending on the mean curvatures $M_a$ and $M_b$ of these two spheres, four different two-sphere morphologies can be distinguished as shown in Fig. 3. The morphologies with out-budded domains have positive *ab*-necks, those with in-budded domains have negative *ab*-necks. The stability of the four two-sphere morphologies as formed by two-domain vesicles depends on the stability of their closed *ab*-necks. The corresponding stability relations are given by Eq. (44) for positive *ab*-necks and by Eq. (55) for negative *ab*-necks. The resulting morphology diagrams are displayed in panels a and b of Fig. 6.

The closed *ab*-necks experience constriction forces as defined by Eq. (63), which act to compress these necks. The form of the constriction forces is provided by Eq. (64) for out-budded domains with positive *ab*-necks and by Eq. (67) for in-budded domains with negative *ab*-necks. These constriction forces must exceed about 20 pN in order to induce membrane fission across the closed neck [33], thereby dividing the budded vesicle into two daughter vesicles. It is argued in Sect. 4.3 that the membrane necks undergo fission for large line tensions of the domain boundaries and/or large spontaneous curvatures but remain stable against fission for smaller line tensions and moderate spontaneous curvatures. If the Gaussian curvature moduli of the *a*- and *b*-domains are different, the constriction forces are given by Eqs. (98) and (99), which contain no contribution from the line tenion of the domain boundary. As a consequence, different Gaussian curvature moduli $\kappa _{Gb} \ne \kappa _{Ga}$ act to reduce the constriction forces at closed membrane necks, see Sect. 10.2.

The morphological complexity of multispherical shapes formed by multi-domain vesicles arises from two different mechanisms. First, each domain of a two-domain vesicle with a single *ab*-neck can form a multispherical shape by itself. Second, vesicles with more than two domains can form multispheres with more than one *ab*-neck. Examples for multispherical shapes with one and several *ab*-necks are displayed in Figs. 7 and 8, respectively. In addition to the *ab*-necks, these shapes involve closed *aa*- and *bb*-necks. The stability regimes for the latter necks are displayed in Fig. 9. In general, all necks of a multisphere must be stably closed, a condition that acts to reduce the stability regime of the respective multisphere as illustrated in Figs. 10 and 11. Particularly interesting multispheres are formed by vesicles with nested *a*- and *b*-domains as shown in Fig. 12.

From a mathematical point of view, multispheres represent generalized CMC surfaces, which exhibit up to four different piece-wise constant mean curvatures as discussed in Sect. 8. Examples for conventional CMC surfaces with one constant mean curvature are provided by multispheres consisting of equally sized spheres as in Fig. 2g and by the multispherical triunduloid in Fig. 13c. The latter shape provides a model for the three-way junctions of membrane nanotubes as observed in the endoplasmic reticulum [48]. As explained in Sect. 9, the multispherical shapes obtained here from the theory of curvature elasticity can be studied experimentally, generalizing available protocols for the multisphere formation of uniform membranes, for the fine-tuning of the spontaneous curvatures, and for the preparation of multidomain vesicles by electrofusion.

Two-domain vesicles have also been studied on the nanoscale by simulations using dissipative particle dynamics [49–52]. One of these studies provided a series of simulation snapshots for the closure of the *ab*-neck [52]. The snapshots indicate that the *ab* domain boundary stayed in the membrane neck during the whole neck closure process of the nanovesicle. It then follows from Eq. (40) and Sect. 10 that the two Gaussian curvature moduli $\kappa _{Ga}$ and $\kappa _{Gb}$ were identical for the *a*- and *b*-domains of the vesicles studied in Ref. [52]. Additional simulation studies are required in order to determine the location of the domain boundary during the neck closure process for other two-domain vesicles. Based on recent simulation results for nanovesicles [53], one would expect that the difference in the two Gaussian curvature moduli will depend on the stress asymmetry between the leaflet tensions of the lipid bilayers.

## Data Availability

My manuscript has no associated data.

## Appendix A: Theory of two-domain vesicles

In the theoretical description used here, we ignore the width of the domain boundary between the *a*- and *b*-domains. This simplification is justified when the linear size of the *a*- and *b*-domains is large compared to the boundary width, a condition that is usually fulfilled for the optically resolvable membrane domains of giant vesicles.^4^ Because we ignore the width of the domain boundary, the bending rigidity and the spontaneous curvature change abruptly as we cross this boundary. Nevertheless, we can still impose the physical requirement that the shapes of the two membrane domains meet ‘smoothly’ along the domain boundary, i.e., that these shapes have a common tangent along this boundary, as explicitly shown for axisymmetric vesicle shapes [23].

### A.1 Elastic energy of two-domain vesicle

The elastic energy of a membrane segment depends on its mean curvature *M* and on its Gaussian curvature *G*. These curvatures are defined in terms of the two principal curvatures $C_1$ and $C_2$ according to

A1 $$\begin{aligned} M = \frac{1}{2} \left( C_1 + C_2 \right) \quad \textrm{and} \quad G = C_1 C_2 \end{aligned}$$

Now, consider a vesicle of volume *V* that is bounded by a membrane with one *a*-domain and one *b*-domain. We can then decompose the vesicle shape *S* into three components: the shapes $S_a$ and $S_b$ of the two domains as well as the shape $S_{ab}$ of the domain boundary. The *a*- and *b*- domains have the surface areas $A_a$ and $A_b$, the domain boundary between the *a*- and *b*-domain has the length $L_\textrm{db}$.

The elastic energy of such a two-domain vesicle can be decomposed into several contributions: the curvature energy of the *a*-domain, the curvature energy of the *b*-domain, and the line energy of the *ab* domain boundary. The curvature energies can be further decomposed into bending and Gaussian curvature contributions. The bending energy $E_\textrm{be}\{ S_a\}$ of the *a*-domain depends on the (local) mean curvature *M*, the spontaneous curvature $m_a$, and the bending rigidity $\kappa _a$. Likewise, the bending energy $E_\textrm{be}\{ S_b\}$ of the *b*-domain depends on the (local) mean curvature *M*, the spontaneous curvature $m_b$ and the bending rigidity $\kappa _b$. These bending energies have the form [23, 31]

A2 $$\begin{aligned} E_\textrm{be}\{ S_a\} = \int \! \textrm{d}A_a \, 2 \kappa _a (M - m_a)^2 \end{aligned}$$

and

A3 $$\begin{aligned} E_\textrm{be}\{ S_b\} = \int \! \textrm{d}A_b \, 2 \kappa _b (M - m_b)^2 \,, \end{aligned}$$

which generalizes the spontaneous curvature model for a uniform membrane [29, 54] to the case of two different intramembrane domains. The Gaussian curvature energy of the two domains is given by

A4 $$\begin{aligned} E_G \{ S_a, S_b \} \equiv \kappa _{G a} \int \! \textrm{d}A_a \, G + \kappa _{G b} \int \! \textrm{d}A_b \, G \end{aligned}$$

which depends on the two Gaussian curvature moduli $\kappa _{Ga}$ and $\kappa _{Gb}$ of the *a*- and *b*-domains. For an axisymmetric shape, the energy in Eq. (A4) can be transformed into $E_G \{ S_a, S_b \} = 2 \pi \left( \kappa _{Ga} + \kappa _{Gb} \right) + \Delta E_G$ with [23]

A5 $$\begin{aligned} \Delta E_G = 2 \pi \left( \kappa _{Ga} - \kappa _{Gb} \right) \cos \psi (s_\textrm{db}) \end{aligned}$$

where $\psi (s_\textrm{db})$ is the tilt angle $\psi$ of the shape contour at the domain boundary with arc length $s_\textrm{db}$.

The energy $E_{2 \textrm{Do}}$ of the two-domain vesicle is then obtained by summing up the different energy contributions which leads to [23, 31]

A6 $$\begin{aligned} E_{2 \textrm{Do}}{ \{ S \} }= E_\textrm{be}\{ S_a\} + E_\textrm{be}\{ S_b \} + E_G \{ S_a, S_b \} + \lambda \, L_\textrm{db}\nonumber \\ \end{aligned}$$

where the last term represents the energy contribution from the domain boundary which is proportional to the line tension $\lambda$ [22]. Stable domain patterns imply positive line tensions, $\lambda > 0$.

### A.2 Shape functional for two-domain vesicles

The equilibrium shapes of a two-domain vesicle are obtained by minimizing the energy in Eq. (A6) imposing the constraints of a certain vesicle volume *V* as well as certain areas membrane $A_a$ and $A_b$ of the *a*- and *b*-domains. These three constraints can be taken into account by three Lagrange multipliers ${\Delta \!P}$, $\Sigma _a$, and $\Sigma _b$. As a consequence, the shape functional of the two-domain vesicle has the form

A7 $$\begin{aligned} F_{2 \textrm{Do}}{ \{ S \} }= - {\Delta \!P}\, V + \Sigma _a \, A_a + \Sigma _b \, A_b+ E_{2 \textrm{Do}}{ \{ S \} }\end{aligned}$$

with the energy $E_{2 \textrm{Do}}$ as given by Eq. (A6). So far, a systematic minimization of this functional has been performed for axisymmetric vesicles using the shooting method for the integration of the shape equations [23] and, to some extent, by numerical minimization of discretized membranes [34]. In these numerical studies, the spontaneous curvatures were taken to be relatively small. The same energy has also be used to calculate doubly-periodic bicontinuous shapes corresponding to ‘lattices of passages’ [55].

## Appendix B: Glossary

### Abbreviations

Chol: Cholesterol
CMC: Constant-mean-curvature
DOPC: Phospholipid 1,2-dioleoyl-sn-glycero-3-phosphocholine
GFP: Green fluorescent protein
GUV: Giant unilamellar vesicle, abbreviated as “giant vesicle”
Ld: Liquid-disordered lipid phase
Lo: Liquid-ordered lipid phase
POPC: Phospholipid 1-palmitoyl-2-oleoyl-sn-glycero-3-phosphatidylcholine
POPG: Phospholipid 1-palmitoyl-2-oleoyl-sn-glycero-3-phospho-(1’-rac-glycerol)
SM: Spingomyelin

### Mathematical symbols

The following list is ordered alphabetically, with Greek letters treated as words.

*a*, *b*: Domain labels for two distinct intramembrane domains
$a_k$: Individual *a*-domain labeled by integer *k*
$a_{ki}$: Individual *a*-sphere formed by $a_k$-domain and labeled by *i*
*A*: Surface area of vesicle membrane
$A_a$: Surface area of *a*-domain
$A_{ak}$: Surface area of $a_k$-domain
$A_b$: Surface area of *b*-domain
$A_{bn}$: Surface area of $b_n$-domain
$b_n$: Individual *b*-domain labeled by integer *n*
$b_{nj}$: Individual *b*-sphere formed by $b_n$-domain and labeled by *j*
$C_{1,\textrm{wl}}$: First principal curvature perpendicular to the waist-line of an open neck
$C_{2,\textrm{wl}}$: Second principal curvature parallel to the waist-line of an open neck
$E_\textrm{be}$: Bending energy of membrane
*f*: Constriction force at closed membrane neck
*G*: Gaussian curvature
$h_\textrm{out}(x)$: Linear function of *x* for out-budded limit shapes $L_{ab}$
$h_{a\text {-in}}(x)$: Linear function of *x* for limit shapes $L_{ab}$ with in-budded *a*-domains
$h_{b\text {-in}}(x)$: Linear function of *x* for limit shapes $L_{ab}$ with in-budded *b*-domains
*i*, *j*: Integer labels for individual spheres
*k*: Integer label for different *a*-domains
$\kappa$: Bending rigidity of membrane
$\kappa _a$: Bending rigidity of *a*-domain
$\kappa _b$: Bending rigidity of *b*-domain
$\kappa _{G}$: Gaussian curvature modulus
$\kappa _{G a}$: Gaussian curvature modulus of *a*-domain
$\kappa _{G b}$: Gaussian curvature modulus of *b*-domain
$L_{ab}$: Limit shape for multispherical shape with closed *ab*-neck
$\lambda$: Line tension of domain boundary
*m*: Spontaneous curvature of membrane
${\bar{m}}$: Rescaled spontaneous curvature, ${\bar{m}}= m R_\textrm{ve}$
$m_a$: Spontaneous curvature of *a*-domain
$m_b$: Spontaneous curvature of *b*-domain
*M*: Mean curvature of spherical segment
${\bar{M}}$: Rescaled mean curvature, ${\bar{M}}= M R_\textrm{ve}$
$M_a$: Mean curvature of *a*-sphere
$M_b$: Mean curvature of *b*-sphere
$M_{aa}^\textrm{eff}$: Effective mean curvature of *aa*-neck
$M_{bb}^\textrm{eff}$: Effective mean curvature of *bb*-neck
$M_{ab}^\textrm{eff}$: Effective mean curvature of *ab*-neck
*n*: Integer label for different *b*-domains
$\Phi _a$: Area fraction of *a*-domains
$\Phi _b$: Area faction of *b*-domains
$\psi$: Tilt angle of surface normal for axisymmetric shape
$\psi (s_\textrm{db})$: Tilt angle of surface normal at domain boundary
*R*: Curvature radius of spherical segment, which is always positive
*r*: Rescaled curvature radius of spherical segment, $r \equiv R/R_\textrm{ve}$
$R_a$: Radius of *a*-sphere for two-sphere vesicles
$R_{aki}$: Radius of *a*-sphere formed by $a_k$-domain
$R_{ak1}$: Radius of *a*-sphere formed by $a_k$-domain and connected to *b*-sphere
$R_b$: Radius of *b*-sphere for two-sphere vesicles
$R_{bnj}$: Radius of *b*-sphere formed by $b_n$-domain
$R_{bn1}$: Radius of *b*-sphere formed by $b_n$-domain and connected to *a*-sphere
$R_\textrm{db}$: Radius of circular domain boundary
$R_\textrm{ne}$: Radius of circular membrane neck
$R_\textrm{ve}$: Basic length scale provided by vesicle size, $R_\textrm{ve}= \sqrt{A/(4 \pi )}$
*s*: Arc length for contour of axisymmetric shape
$s_\textrm{db}$: Arc length for domain boundary
*V*: Vesicle volume
*v*: Rescaled volume, $$v = V/ ( \frac{4 \pi }{3} R_\textrm{ve}^3)$$
*x*: Coordinate of morphology diagrams with $x = {\bar{m}}_a$
*y*: Coordinate of morphology diagrams with $y = {\bar{m}}_b$

## Footnotes

1 A stably closed neck can also be formed between a spherical *b*-domain and a prolate *a*-domain [23]. In the latter case, the local stability condition in Eq. (44) is still valid but the mean curvatures can no longer be expressed in terms of the sphere radii.

2 In principle, one may also consider a situation with constrained equilibrium in which each $a_k$-domain has a conserved membrane area $A_{ak}$. In the latter situation, the term $\Sigma _a \sum _k A_{ak}$ in Eq. (70) is replaced by $\sum _k \Sigma _{ak} A_{ak}$, which implies that each $a_k$-domain is governed by a different shape equation.

3 This ordering of the Gaussian curvature moduli agrees with Fig. 11 of Ref.  [23] but disagrees with two misleading statements in the main text of this reference, as pointed out by Tobias Baumgart.
